# Circulating extracellular vesicles expressing PD1 and PD-L1 predict response and mediate resistance to checkpoint inhibitors immunotherapy in metastatic melanoma

**DOI:** 10.1186/s12943-021-01490-9

**Published:** 2022-01-18

**Authors:** Simona Serratì, Michele Guida, Roberta Di Fonte, Simona De Summa, Sabino Strippoli, Rosa Maria Iacobazzi, Alessandra Quarta, Ivana De Risi, Gabriella Guida, Angelo Paradiso, Letizia Porcelli, Amalia Azzariti

**Affiliations:** 1 Laboratory of Nanotechnology, IRCCS Istituto Tumori Giovanni Paolo II, V.le O. Flacco, 65, 70124 Bari, Italy; 2 Rare Tumors and Melanoma Unit, IRCCS Istituto Tumori Giovanni Paolo II, V.le O. Flacco, 65, 70124 Bari, Italy; 3 Laboratory of Experimental Pharmacology, IRCCS Istituto Tumori Giovanni Paolo II, V.le O. Flacco, 65, 70124 Bari, Italy; 4 Molecular Diagnostics and Pharmacogenetics Unit, IRCCS Istituto Tumori Giovanni Paolo II, V.le O. Flacco, 65, 70124 Bari, Italy; 5 CNR NANOTEC-Istituto di Nanotecnologia, National Research Council (CNR), via Monteroni, 73100 Lecce, Italy; 6 Department of Basic Medical Sciences Neurosciences and Sense Organs, University of Bari, Piazza G. Cesare, 11, 70124 Bari, Italy; 7 Scientific Directorate, IRCCS Istituto Tumori Giovanni Paolo II, V.le O. Flacco, 65, 70124 Bari, Italy

**Keywords:** Metastatic melanoma, Drug resistance, Extracellular vesicles, PD1, PD-L1, Anti-PD1 treatment

## Abstract

**Background:**

The immunotherapy with immune checkpoints inhibitors (ICI) has changed the life expectancy in metastatic melanoma (MM) patients. Nevertheless, several patients do not respond hence, the identification and validation of novel biomarkers of response to ICI is of crucial importance. Circulating extracellular vesicles (EVs) such as PD-L1^+^ EV mediate resistance to anti-PD1, instead the role of PD1^+^ EV is not fully understood.

**Methods:**

We isolated the circulating EVs from the plasma of an observational cohort study of 71 metastatic melanoma patients and correlated the amount of PD-L1^+^ EVs and PD1^+ ^EVs with the response to ICI. The analysis was performed according to the origin of EVs from the tumor and the immune cells. Subsequently, we analysed the data in a validation cohort of 22 MM patients to assess the reliability of identified EV-based biomarkers. Additionally we assessed the involvement of PD1^+^ EVs in the seizure of nivolumab and in the perturbation of immune cells-mediated killing of melanoma spheroids.

**Results:**

The level of PD-L1^+^ EVs released from melanoma and CD8^+ ^T cells and that of PD1^+^ EVs irrespective of the cellular origin were higher in non-responders. The Kaplan-Meier curves indicated that higher levels of PD1+ EVs were significantly correlated with poorer progression-free survival (PFS) and overall survival (OS). Significant correlations were found for PD-L1^+^ EVs only when released from melanoma and T cells. The multivariate analysis showed that high level of PD1^+^ EVs, from T cells and B cells, and high level of PD-L1^+^ EVs from melanoma cells, are independent biomarkers of response. The reliability of PD-L1^+^ EVs from melanoma and PD1^+^ EVs from T cells in predicting PFS was confirmed in the validation cohort through the univariate Cox-hazard regression analysis. Moreover we discovered that the circulating EVs captured nivolumab and reduced the T cells trafficking and tumor spheroids killing.

**Conclusion:**

Our study identified circulating PD1^+^ EVs as driver of resistance to anti-PD1, and highlighted that the analysis of single EV population by liquid biopsy is a promising tool to stratify MM patients for immunotherapy.

**Supplementary Information:**

The online version contains supplementary material available at 10.1186/s12943-021-01490-9.

## Background

Before the advent of BRAF/MEK targeted therapy and immune checkpoint inhibitors (ICI), metastatic melanoma (MM) was marked by a poor prognosis with an overall survival of 8-10 months [1]. Nowadays, the 30% long-term survivors with anti-PD1 monotherapy and the 50% with anti-CTLA4 plus anti-PD1 combination, are emblematic of how immunotherapy has changed the prognostic landscape of MM [2]. However many questions have arisen, mainly focusing on patient’s selection for immunotherapy, since almost 60% of them develop resistance and several don’t respond at all to ICI for reasons that are not yet understood. To date, patient’s selection for immunotherapy is based on the PD-L1 expression [3] and on the genomic assessment of tumor mutational burden [4]. Despite both are predictors of a better response to ICI, they are not fully satisfactory because such evaluations are not effective in capturing the dynamic spatial and temporal heterogeneity of the tumor [5] and additionally they return indistinct antigenic value of individual mutations [6]. Ideally, the identification of blood circulating markers could represent a helpful strategy to fulfil the need to reach a minimally invasive and easily handy tool for both predicting therapeutic outcomes and real-time monitoring disease progression and acquisition of ICI resistance. In terms of candidate predictors, growing interest has been focusing on the exploitation of circulating extracellular vesicles (EVs), released by both normal and cancer cells for the clinical assessment of patients [7, 8].

Circulating EVs are bilayer lipid membrane vesicles, including apoptotic bodies, exosomes and microvesicles carrying a rich molecular array resembling their parental origin, that are involved in cellular cross-talk, shaping of tumor microenvironment and immune escape [9–14]. Increasing evidence showed that circulating EVs may counter antitumor immunity systemically since checkpoint ligands, such as PD-L1, CTLA4, and NKG2D are expressed on their surface [15–18]. Due to their properties, EVs are extensively investigated in melanoma [19] and increasing data indicate that they are predictive biomarker for immunotherapy efficacy [20–22], since they play a role in ICI resistance mechanisms [23, 24]. Accordingly, we discovered that a significant increase of circulating uPAR^+^ (urokinase-type Plasminogen Activator Receptor) EVs released from melanoma, CD8^+^ T-cells and dendritic cells correlated with unresponsiveness in a cohort of MM patients subsequently treated with nivolumab or pembrolizumab, further supporting the notion that EV-based biomarkers are powerful tool to predict innate resistance to ICI [25].

Recently, Machiraju demonstrated the existence of a negative correlation between the patient’s outcome to ICI treatment and the levels of soluble circulating PD1 [26], however the significance of circulating PD1 remains not fully understood. Owing the evidence that the EVs are the main source of circulating PD1 [24], we sought to understand in the same cohort of MM patients, whose data are reported in [25], whether PD1 is expressed on the membrane of circulating EVs and if a putative systemic increase of PD1^+^ EVs in plasma of patients may cause or correlates with unresponsiveness to anti-PD1. Additionally, we performed the evaluation of PD-L1 expression on circulating EVs, for gaining insights on the influence of EV-based expression of PD1/PDL-1 immune checkpoint axis in predicting response to ICI. To evaluate the predictive potential of PD1^+^ EVs and PDL-1^+^ EVs, we correlated the percentage of such EVs in the plasma of patients with their outcomes to ICI treatment. However, given our previous study showing that, rather than the burden of circulating EVs, it was the parsing of EVs populations for their parental origin which allowed to discriminate between responders and non-responders to ICI [25], we performed the correlations by considering the percentage of PD1^+^ EVs and PDL-1^+^ EVs released from both tumor and non-tumor cells (immune cells) in the multivariate analysis. To evaluate if the PD1^+^ EVs may cause unresponsiveness to anti-PD1, we performed in vitro experiments to evaluate if they were involved in the seizure of the therapeutic monoclonal antibodies nivolumab and, if the circulating EVs impacted on immune cells-mediated killing of melanoma spheroids, to demonstrate that they are mediators of treatment failure. Finally, we validated the independent factors identified in the multivariate analysis as biomarkers of response in a validation cohort of 9 responders and 13 non-responders recently enrolled in our study.

## Methods

### Patients and study design

In this observational cohort study we retrospectively recruited 71 patients with stage IV melanoma treated with PD1 inhibitors (nivolumab or pembrolizumab) alone or in combination with ipilimumab according to their standard schedules. The collected baseline clinical features accounted age, sex, type of melanoma, M stage, number of metastatic sites, BRAF status, ECOG (Eastern Cooperative Oncology Group Performance Status), LDH value, neutrophil to lymphocyte ratio (NLR) and platelet to lymphocyte ratio (PLR). Peripheral blood samples were collected at baseline (the day of the first cycle) as well as the radiological assessment that was repeated every 3 months by CT or MRI scan using the immune response criteria for solid tumours (iRECIST) [27] as evaluating tool. We determined the progression free survival (PFS), the overall survival (OS), the objected response rate as the sum of complete (CR) and partial response (PR) and the disease control rate (DCR) including stable disease (SD) lasting more than 6 months. Patients were enrolled in the study from March 2017 to October 2019, follow up continued until patients died or until the final update in September 2020 (median follow-up of 11 months). The study was approved by the local Ethics Committee of Istituto Tumori Giovanni Paolo II in Bari (prot. n. 590/16 EC) and was conducted in accordance with the international standards of good clinical practice. In the validation independent cohort, we prospectively recruited 22 MM patients all treated with anti-PD1. The patient and disease characteristics of this validation cohort were quite similar to those of the observational one as reported in Supplemental Table 1S.

### Blood and plasma sample collection

All samples were acquired through collection of peripheral blood in sodium citrate tubes from MM patients before immunotherapy (71 + 22 patients) and from 3 healthy donors. After the first blood withdrawal for routine analysis, the subsequent blood samples were utilized for EVs isolation. The withdrawal was performed from the arm using a tourniquet, which didn’t impact on the EV isolation as reported by [28], in the morning (8-10 a.m.) and on an empty stomach. Following 30 min incubation at 25 °C after blood collection, the samples were centrifuged at 2500 rpm for 15 min.

### EVs-depleted plasma preparation

Fresh plasma collected from healthy donors was centrifuged at 300 x g for 10 min, at 2,000 x g for 10 min, and then at 10,000 x g for 30 min in order to remove the dead cells and the cell debris, respectively. The supernatant was ultracentrifuged at 100,000 x g for 70 min. The resulting EVs-depleted plasma was utilized for the functional studies described below.

### PBMC isolation

PBMCs were separated after gradient centrifugation of the blood over Ficoll-Hypaque density gradient from 5 to 8 ml of peripheral blood as previously described [29]. The isolated cells were stored at − 195 °C.

### EVs isolation

Following MISEV18 line guides [30], EVs were isolated by ultracentrifugation according to the Thery protocol [31]. In detail, 5 ml of fresh plasma were centrifuged at 2,600 x g for 15 min as previously described [31, 32]. The supernatant were diluted 1:1 in PBS and filtered with 200-nm pore size filters. The resulting plasma was ultracentrifuged at 10,000 x g for 30 min and then twice at 100,000×g for 70 min and the pellet was resuspended in PBS. The pooled EVs, in aliquots, were stored at − 80 °C in order to avoid freezing-thawing to preserve the EV integrity and quantity [30, 33].

### Nanoparticle tracking analysis (NTA)

According to MISEV18 line guides for EV characterization [30], samples were analyzed with the NanoSight NS300 (Malvern Panalytical) following the manufacturer’s instructions (NanoSight NS300 User Manual, MAN0541-02-EN, 2018) [34]. A volume of 5-10 μL of each EV sample were properly diluted and the flowing particles were recorded at constant syringe flow (flow rate = 50) using the sCMOS camera. In particular three 60-s videos were acquired and all measurement were carried out as previously described [25].

### Transmission electron microscopy (TEM) imaging

A drop of EVs suspension was deposited onto a lacey carbon coated copper TEM grid, 300 mesh. Then, the grid was stained with 1% osmium tetroxide for 1 min prior to be rinsed with ultrapure water and let to dry. Low-magnification images were recorded on a JEOL Jem1011 microscope (Tokyo, Japan) operating at an accelerating voltage of 100 kV.

### Dynamic light scattering (DLS) analysis

A suspension of EVs containing 0.01% Triton was transferred into a glass cuvette. The measures were performed at 25 °C using a Zetasizer Nano ZS90 (Malvern Instruments Ldt) equipped with a 4.0 mW He–Ne laser operating at 633 nm and with an avalanche photodiode detector. At least three measures for each sample were performed.

### PBMCs characterization by flow cytometry (FCM)

After thawing, the isolated PBMCs were incubated with the Super Bright Complete Staining Buffer (eBiosciences, Invitrogen), according to the manufacturer’s instructions, and afterwards the cells were labelled with the anti-human antibodies. After staining, the PBMCs were washed with 1xPBS, free of Ca_2_^+^ and Mg_2_^+^, (DPBS), collected and analyzed using an Attune TMNxT Acoustic Focusing Cytometer (Thermo Fisher) equipped with four lasers (405 nm violet, 488 nm blue, 561 nm yellow, and 637 nm red) for the samples reading. The data were analyzed with the Attune TMNxT Analysis Software (Thermo Fisher) as previously described [25].

### EVs characterization by FCM

The FCM instrument preparation and setup was performed as described in M&M S1 and reported [25, 35, 36]. The EVs samples were incubated with 5 μl of Super Bright Complete Staining Buffer (E-Biosciences, Invitrogen) for 30 min at 4 °C, as reported above. Then the EVs were labelled with the anti-human antibodies and stored for 30 min in a dark room at 2 − 8 °C. Finally, EVs were collected and analyzed using an Attune TMNxT Acoustic Focusing Cytometer (Thermo Fisher) as described above.

### Primary labelled antibodies

Primary labelled antibodies were obtained by eBiosciences (Thermo Fisher Scientific, Waltham, MA USA): anti-human-CD9 (FITC, Clone: eBioSN4, SN4 C3-3A2) (0.125 μg/test), anti-human-CD63 (PE-CYN7, Clone: H5C6) (0.5 μg/test), anti-human-CD81 (APC, Clone: 1D6) (1 μg/test), anti-human-CD146 (PE, Clone: P1H12) (0.125 μg/test), anti-human-CD1a (eFluor-450, Clone: H149) (0.5 μg/test), anti-human-CD8 (PE-CYN5, Clone: RPA-T8) (0.25 μg/test), anti-human-CD14 (PE-EF610, Clone: 61D3) (0.25 μg/test), anti-human-CD19 (EF506, Clone: HIB19) (0.5 μg/test), anti-human-CD274 (PD-L1, B7-H1) (Alexa Fluor® 700, Clone: MIH1) (1 μg/test), anti-human-CD279 (PD1) (Super Bright 600, Clone:eBioJ105, J105) (0.5 μg/test).

### Labelling of PBMCs and EVs

According to the manufacturer’s instructions, both EVs isolated from plasma and PBMCs were labelled using PKH26 Red Fluorescent Cell Linker kit and PKH67 Green Fluorescent Cell Linker kit (Sigma-Aldrich, St. Louis, MO), respectively.

### LND1 spheroid formation and PBMCs trafficking evaluation

The human melanoma cells LND1 were grown in high-glucose Dulbecco’s modified Eagle’s medium (DMEM) supplemented with 10% (v/v) foetal bovine serum (FBS), 1% (v/v) L-glutamine, 1% (v/v) penicillin/streptomycin, at 37 °C in a humidified atmosphere at 5% CO_2_. 4 × 10^4^ cells/well were seeded dispensing 1 ml/well of culture medium supplemented with 10% of exosome-depleted FBS (FBS South America, exosome depleted, Bio West, France), into a 24-well flat-bottomed non-treated plate. The plate was then transferred to an incubator (37 °C, 5% CO_2_). Two days later, the LND1 spheroids (about 1 × 10^5^cells) were treated with 1 × 10^5^ PKH26 green-PBMCs, preincubated for 1 h with 100 μg PKH67 red-EVs [37], with or without 20 μg/ml nivolumab (Selleck Chemical, Selleck USA). Each experimental condition was in triplicate. After 2 h and 24 h, cells were analyzed using a fluorescence microscope fitted with a digital camera (Leica DMi8, Leica Microsystem Imaging Solutions Ltd., Cambridge, UK) and images collected.

### Tumour cell killing assay

To evaluate the killing activity, 1 × 10^5^ PBMCs isolated from healthy donors were preincubated with 100 μg EVs from responders and non-responders. Then, we preformed the co-cultured of the 3D tumour colonies (LND-1 spheroids) with pretreated PBMCs (ratio 1:1) in presence or absence of 20 μg/ml nivolumab (Selleck Chemical, Selleck USA) for 96 h. At the end of the co-incubation, we evaluated the percentage of died melanoma cells by FCM using the Fixable Viability Dye eFluor™ 780 (Thermo Fisher Scientific, Waltham, MA USA).

### Fluorescent nivolumab and its binding to circulating EVs

In order to evaluate the binding between circulating EVs and the anti-PD1 nivolumab, we firstly conjugated the anti-PD1 with a fluorescent tag (fluo-nivolumab). Briefly, 300 μg nivolumab (Selleck Chemical, Selleck USA) prior to modification, was purified from storage buffer including excess azide reagent using 50 kDa Amicon filters (EMD Millipore, Burlington, MA). Antibody modification was performed using the SiteClick™ Antibody Azido modification kit (Thermo Fisher Scientific, Waltham, MA USA) following the manufacturer’s instructions. After purification, the absorbance of the azido-modified nivolumab was measured on amicroplate reader (MULTISKAN Sky, Thermo Scientific). The maximum absorbance of the antibody was measured at 280/260 nm. Then, 100 μg azido-modified nivolumab was labelled using the Click-iT™ Alexa Fluor™ 555 sDIBO Alkyne for SiteClick™ Antibody Labeling kit (Thermo Fisher Scientific, Waltham, MA USA) to obtain fluo-nivolumab according to the protocol provided by the manufacturer.

The EVs from 6 MM patients were incubated with the obtained fluo-nivolumab at the same concentration of the commercial anti-PD1 antibody (0.5 μg/test). The FCM analysis was performed as described above, utilising anti-CD9, anti-CD63 and anti-CD81 to discriminate EVs. Experiments were carried out in DPBS or in EVs-depleted plasma from healthy donors.

### Statistical analysis

Statistical significance was calculated using two-tailed t-tests, analysis of variance, Kruskal-Wallis tests, Dunn tests, Mann-Whitney U tests and two-tailed ANOVA. Statistical significance was set at *p* < 0.05 (*p < 0.05, **p < 0.01, and ***p < 0.001). Statistical analyses were performed using GraphPad Prism V.5.0 software (GraphPad Software, San Diego, California, USA). Survival analyses and test for equality of proportions has been performed through R v.3.6.3 environment. In detail, Kaplan-Meier curves and Cox hazard regression analyses were implemented by “survival” package. Mantel-Cox test to compare Kaplan-Meier curves has been performed by “survminer” package. “ggplot2” package was used to depict survival curves, multivariate Cox-hazard regression analysis (“forestmodel” package) and barplots. “survival ROC” package has been used to evaluate the performance of multivariate Cox hazard regression models.

## Results

### Clinical outcomes

Collectively, we enrolled 71 and 22 patients with MM who have been treated with ICI therapy in the observational and in the validation studies, respectively. Observational cohort: patient characteristics and outcomes to ICI therapy are summarised in Table 1, whose data are already reported in [25] in which the same series of patients were screened to profile the circulating uPAR^+^ EVs. Briefly, we reported 29 (40.8%) responses with 8 complete. The DCR including CR, PR and SD lasting more than 6 months was 46,5% (*n* = 33), whereas the PD rate was 53.5% (*n* = 38). The median PFS and the median OS of the entire population was 4 and 11 months, respectively. Validation cohort: patient characteristics and outcomes to ICI therapy are summarised in Supplemental Table S1.

**Table 1:** Patients information. Clinical characteristics and outcomes to anti PD1 treatment of the study cohort (*n* = 71)

**Age**	60 (33-86)
**Sex, n (%)**	
male	35 (49.3)
female	36 (50.7)
**Type of melanoma, n (%)**	
Cutaneous	51 (71.8)
Uveal	6 (8.4)
Mucosal	3 (4.2)
Unknown origin	12 (16,9)
**BRAF Status, n (%)**	
mutated	34 (47.9)
wt	37 (52.1)
**Previous systemic therapy for metastatic disease, n (%)**	
yes	34 (47.9)
no	37 (52.1)
**Stage at metastatic disease, n (%)**	
M1a	14 (19.7)
M1b	9 (12.7)
M1c	31 (43.7)
M1d	17 (23.9)
**N. of metastatic sites, n (%)**	
< 3	39 (54.9)
≧3	32 (45.1)
**PS (ECOG), n (%)**	
0	32 (45.1)
1	33 (46.5)
2	6 (8.4)
**LDH, n (%)**	
1x ULN	35 (49.3)
2x ULN	28 (39.4)
> 2x ULN	7 (9.9)
Unspecified	1 (1.4)
**NLR, median (range)**	2.31 (0.83-13.19)
**PLR, median (range)**	141.88 (54.61-518)
**Best response, n (%)**	
ORR	29 (40.8)
DCR	33 (46.5)
CR	8 (27.6)
PD	38 (53.5)
**PFS median, months**	4
**OS median, months**	11

*ULN* upper limit of normal

### EV isolation and characterisation

**Fig. 1:**
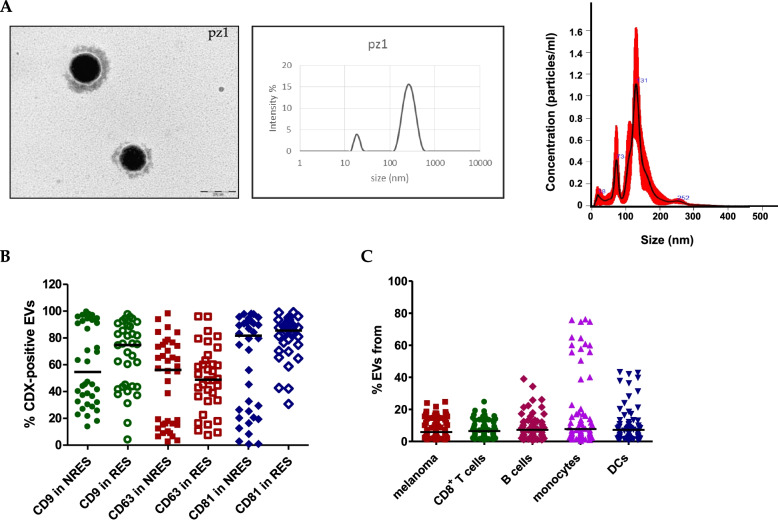
Circulating EVs characterization. **A** TEM images of EVs isolated from plasma of patient 1 (scale bar is 200 nm), DLS plot and NTA histograms with confidence interval in red reporting the concentration and specific particle size of the same EVs. **B** Scatter plot of the percentage of CD9^+^ EVs, CD63^+^ EVs, and CD81^+^ EVs in responders (*n* = 38) and non-responders (*n* = 33) analysed by FCM. **C** Scatter plot with median showing the percentage of EVs derived from melanoma cells (CD146^+^), T cells (CD8^+^) and B cells (CD19^+^), monocytes (CD14^+^) and DC (CD1a^+^)

For each patient before ICI, we collected blood samples to obtain plasma from which we isolated EVs by ultracentrifugation [25, 32]. The EVs were characterised for size and concentration by TEM imaging, DLS and NTA. In Fig. 1a, the characterization of EVs from patient 1 is shown and the morphology appeared proper of EVs with size of about 100 nm by TEM, Z average of 265 ± 35 and PDI of 0.536 by DLS and smaller than 200 nm for more than 80% of the EVs by NTA. The EVs size analysed in TEM images resulted a little smaller than that measured by NTA, but it is worth to remind that under the TEM the EVs are dry and thus shrank and contracted. The histograms and the TEM images in Fig. 1 are representative of all EVs recovered from plasma samples. Therefore, we have generically used the term EVs to denote a mixture of small EVs composed mainly (> 80%) of EVs measuring < 200 nm, compatible with exosomes measuring 40–200 nm [38].

The positivity for three exosomal/EVs markers, such as the tetraspanins CD9, CD63 and CD81, was determined by FCM [39]. CD9^+^, CD63^+^ and CD81^+^ EVs isolated from plasma of responders and non-responders are reported (Fig. 1B). By evaluating the double and triple positivity for the tetraspanins, for each patient we selected EVs which were positive for at least one of the three tetraspanins (see M&M S1). Then, we determined their cellular origin (melanoma cells or immune cells, as T and B lymphocytes, monocytes and dendritic cells) by measuring the percentage of EVs positive for CD146, CD8, CD19, CD14 and CD1a, respectively (Fig. 1C). The choose of CD146 (MCAM) as marker for melanoma, instead of the more common ones, as Pmel-1 and S100 came from a preliminary analysis described in [25]. Our results showed that different origin (cell type) released similar amount of circulating EVs.

### Assessment of PD-L1^+^ EVs in plasma of responders and non-responders

Starting from the importance of PD-L1 expressed by tumor cells in dampening the antitumor immune responses [40, 41], with a negative impact on outcomes in melanoma patients [42], and by reason of the literature results demonstrating that the PD-L1^+^ EVs in the plasma of patients with MM could be a biomarker of ICI resistance [24], we decided to investigate PD-L1^+^ EVs and its cognate PD1^+^ EVs isolated from the plasma of patients with MM before initiating ICI therapy.

**Fig. 2:**
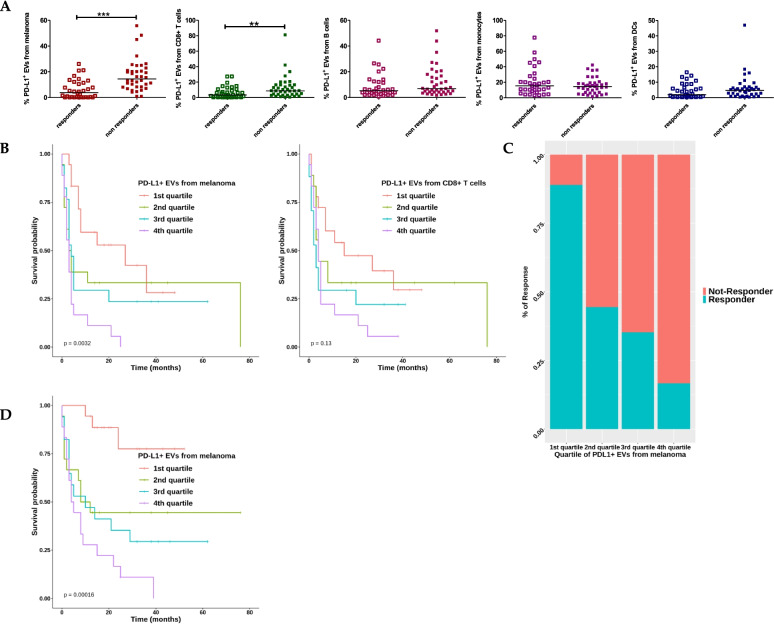
PD-L1^+^ EVs derived from different cell types released clustered by response to therapy and evaluation of PFS and OS in patients with PD-L1^+^ EVs derived from melanoma cells and CD8^+^ cells. **A** Scatter plot with median of the percentage of PD-L1^+^ EVs from responders (*n* = 38) and non-responders (*n* = 33) (Mann Whitney t test**p < 0.01, *** *p* < 0.001). **B** Kaplan–Meier survival curve analysis according to PD-L1^+^ EVs quartiles, with PD-L1^+ ^EVs from melanoma and CD8 T cells as respect to PFS, and **C** the distribution of the best responses stratifying patients by quartiles of PD-L1^+ ^EVs from melanoma cells. **D** Kaplan–Meier survival curve analysis according to PD-L1^+^ EVs quartiles, with PD-L1^+ ^EVs from melanoma cells as respect to OS

As it is known that tumor-secreted exosomes contain PD-L1 presented both on the surface and within exosome particles [16] we preliminarily assessed the percentage of EV, expressing PD-L1 on the surface, demonstrating that these EVs are slightly higher in responders than in non-responders [25]. Focusing on PD-L1^+ ^EVs released by tumor and immune cells, we observed that only EVs from melanoma and CD8^+^ T cells resulted statistically lower levels in responders than in non-responders (Fig. 2A), in agreement with Chen’s evidences [24]. The relative Kaplan-Meier survival curves are shown in Fig. 2B, D and higher levels of melanoma-derived PD-L1^+^ EVs were correlated with a poorer PFS (*p* < 0,0032) and OS (*p* < 0,00016), while those from CD8^+^ T cells only with OS (*p* < 0.00016). Furthermore, the overall response rate (ORR) analysis confirmed the significance of PD-L1^+^ EVs from melanoma as predictive of ICI resistance in non-responders (Fig. 2C).

The data shown so far evidence a strong correlation between higher levels of PD-L1^+^ EVs and the lack of response to ICI when these are released from melanoma cells and CD8^+^ T cells, further supporting Chen’s hypothesis that exhausted T cells, releasing higher amount of PD-L1^+^ EVs, can no longer be reinvigorated by anti-PD1 treatment [24].

### Assessment of PD1^+^ EVs in plasma of responders and non-responders

In order to observe whether there are also PD1^+^ EVs in the plasma that can play an active role in determining the response to ICI, we determined whether this population of EVs was present. The FCM analysis of total EVs positive for PD1 and/or PD-L1, isolated from plasma of the 71 MM patients enrolled in the study and expressing at least one of the 3 tetraspanins (CD9, CD63 and CD81), showed that PD1^+^ EVs were present at high percentage as PD-L1^+^ EVs while the double positive EVs were much less represented (Supplemental Fig. S1A). The median values of EVs positive for PD-L1 and PD1 decreased from 89.1 to 75.3, difference due to 17 of 71 of MM patients in whom the circulating PD1^+^ EVs were less than 10%. Whether PD1^+ ^EV population is categorized into responders and non-responders, the higher level of PD1^+^ EVs is correlated with the a poorer response to ICI and the circulating PD1^+^ EVs, present in a percentage lower than 10%, all belong to the responders’ group (Supplemental Fig. S1B). The median value of PD1^+^PD-L1^+^ EVs was 20.55% (Fig. S1A), indicating that it was only a small subpopulation of the circulating EVs. However, if this subpopulation was stratified in function to ICI response, it was statistically more abundant in non-responders (Fig. S1C) in which it could represent an additional tumor effort to reduce ICI effectiveness.

If we consider the origin of this population of EVs, no statistically differences were found among melanoma cells and CD8 T cells, B cells, monocytes and dendritic cells (DCs) (Supplemental Fig. S1D). However, if we distinguish the PD1^+^ EVs coming from non-responders compared to responders we observed a statistically significant increase in PD1^+^ EVs in the first group evident both if these were released from melanoma cells and from analysed immune cells (Supplemental Fig. S1E). The PD1^+^ EVs median values of the couples responders *vs* non-responders are 4.86 *vs* 22.27, 4.71 *vs* 14.54, 1.18 *vs* 10.86, 6.87 *vs* 22.43 and 5.07 *vs* 21.45 if they come from melanoma cells, CD8^+^ T cells, B cells, monocytes and DCs, respectively.

**Fig. 3:**
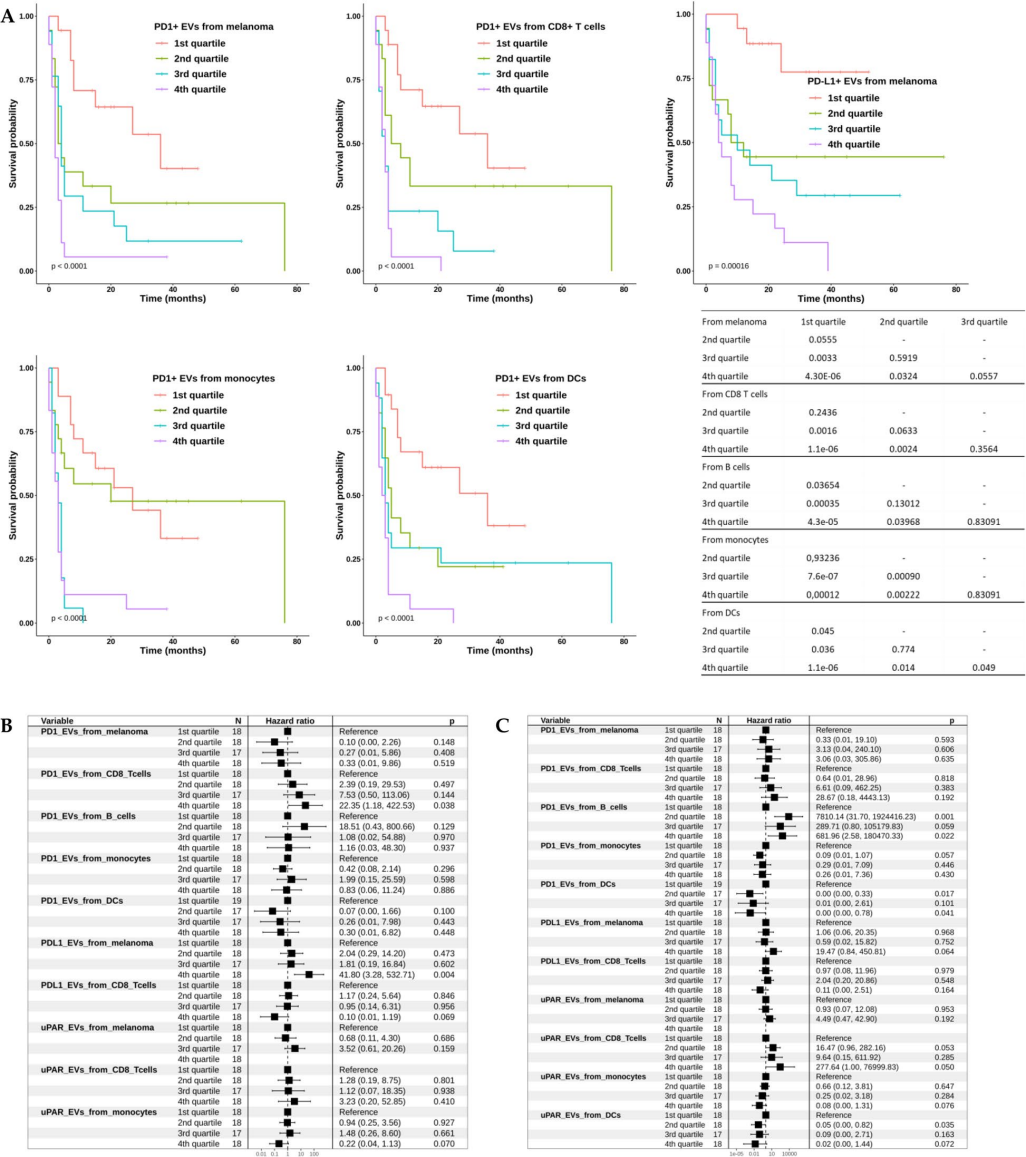
Evaluation of PFS in patients with PD1^+^EVs derived from melanoma cells and immune cells and Multivariate Cox-hazard regression analysis. **A** Kaplan–Meier survival curve analysis according to PD1^+^ EVs quartiles, with these EVs from melanoma cells, CD8^+^ T cells, B cells, monocytes and DCs as respect to PFS . For each analysis, a pairwise comparison of curves has been performed with *p*-values reported in tables next to each graph. Cox analysis for **B** PFS and **C** OS

The evidence that in non-responders *vs* responders quite all cells, among those included in this study, release higher amount of PD1^+^ EVs suggests a promising role for these EV subpopulation to be negative predictors for response to ICI. Thus, we correlated the levels of these EVs of different origins according to their median PFS and OS demonstrating that higher levels of PD1^+^ EVs were strongly correlated with poorer PFS (*p* < 0.0001) and OS (*p* < 0.0001), as shown in the Kaplan–Meier survival curves for the PD1^+^ EV quartiles reported in Fig. 3A and Supplemental Fig. S2.

Furthermore, the overall response rate (ORR) analysis showed that regarding PD1^+^ EVs released from all sources analysed, the proportion of non-responders significantly increased along the quartile stratification (Supplemental Fig. S3). Indeed, in the 1st quartile responder patients are almost 94% passing to almost 1% in the 4th quartile for all the types of PD1^+^ EVs. This evidence has been supported by a strong statistical significance.

Both the dot plot analyses reporting the percentage of EVs in responders and non-responders and the Kaplan-Meier survival curves suggested that these EV subclasses could represent new biomarkers for the ICI response. Therefore, in order to pursue a rigorous analysis for the identification of independent predictive biomarkers, we performed multivariable Cox-hazard regression analyses for both OS and PFS. We also included the data relating to uPAR^+^ EVs analysed in the same series of MM patients and already published in [25]. The analyses were performed both for OS and PFS (Fig. 3B/C). Multivariate analysis included independent variables resulted to be statistically significant in univariate setting (Supplemental Figs. S4, S5). Regarding PFS, PD1^+^ EVs from CD8+ T cells and PD-L1^+^ EVs from melanoma were found to be independent factors related to PFS. In detail, patients in the 4th quartile for their content of PD1^+^ EVs from CD8^+^ T cells and PD-L1^+^ EVs from melanoma showed a higher risk to have a shorter PFS (*p* = 0.038 and *p* = 0.004, respectively). On the contrary, PD-L1^+^ EVs from CD8^+^ T cells and uPAR^+^ EVs from monocytes showed an opposite statistical trend (*p* = 0.069 and *p* = 0.07, respectively). Indeed, patients with the highest content of PD-L1^+^ EVs from CD8^+^ T cells and uPAR^+^ EVs from monocytes have a minor risk to a have a progressive disease. Thus, the Cox analyses on PFS revealed that the most significant variables as negative factors, both in term of hazard ratios and *p*-values, are high content of PD1^+^ EVs from CD8^+^ T cells and of PD-L1^+^ EVs from melanoma (4th quartile HR (95% CI) equal to 22.35 (1.18-422.53) and 41.8 (3.28-532.71), respectively). The performance of the model reveals AUC = 0.86, indicating a strong predictive value.

Regarding OS, we found that PD1^+^ EVs from B cells are independent negative prognostic factors. It could be observed in the forest plot that patients from 2nd to 4th quartile have a greater risk to have a shorter OS. PD-L1^+^ EVs from melanoma and uPAR^+^ EVs from CD8+ T cells showed a statistical trend indicating a negative prognostic role. A positive prognostic role, even if demonstrated only with statistical trends, emerged for PD1^+^ EVs from DCs and uPAR^+^ EVs from monocytes and DCs. Regarding OS, it could be highlighted that the content of PD1^+^ EVs from B cells is the strongest negative prognostic factors. Moreover, the multivariate model has AUC = 0.975, indicating a strong predictive value.

Among the clinical features that we consider in comparison with PD1^+^ EVs levels, we chose LDH, which is a tumour marker accepted as a validated prognostic and predictive factor in MM patients, the BRAF status, the gender, the number of metastatic sites and the pre-treatment of patients with targeted therapy.

The determination of whether PD1^+^ EVs were differently released by MM patients with normal or high LDH level, measured before immunotherapy, evidenced a statistically increased percentage of these EVs only when they were released from melanoma and dendritic cells in patients with high LDH, from a median value of 7.47 to 14.95% and from 8.32 to 15.88%, respectively (Supplemental Fig. S6A). If we consider the possible correlation between PD1^+^ EVs and LDH levels as a function of the response to immunotherapy, non-responders showed a statistically higher level of these circulating EVs irrespective to their origin (cell type), as shown in Supplemental Fig. S6B and Table S2.

The status of the BRAF seems irrelevant with no differences between the patients who harbour BRAF mutation and a wild type gene. As already shown for LDH, non-responders within subgroups with different BRAF status always showed a statistically significant increase in PD1^+^ EVs. The median values are reported in Fig. S6C and Table S2.

Considering the influence of gender [43] and the number of metastasis [44], our analysis showed no differences in the percentage of PD1^+^ EVs with the only exception of those released by DCs which showed a slight increase in responders vs non-responders. Conversely, if we consider the possible correlation between PD1^+^ EVs and these two parameters as a function of the response to immunotherapy, we always found a notable increase in PD1^+^ EVs in the plasma of the non-responders of both subgroups (Supplemental Fig. S6D, Supplemental Fig. S7A and Supplemental Table S2).

As described in Table 1, 34% patients enrolled in this study had undergone previous systemic therapy for metastatic disease. Therefore, we investigated whether the expression of PD1^+^ EVs could change as a function of previous pharmacological treatments. As reported in Supplemental Fig. S7B and Supplemental Table S2, pretreatment with targeted therapy seems not influencing the release of PD1^+^ EVs from the different origin (cell type). However, in each subgroup, we found a strong increase in their release in plasma of non-responders *vs* responders. Finally, no difference was found in the distribution of PD1^+^ EVs as a function of PLR and NLR. However, as already demonstrated for the other parameters, the difference is already statistically significant if in each subgroup, we evaluated the levels of these EVs of any cell origin, present in the plasma of responders and non-responders (Supplemental Fig. S7C/D and Table S2).

All these analyses evidenced that even if the release of total PD1^+^ EVs don’t depend from intrinsic features of the patients, such as gender, LDH, NLR, PLR nor from tumor-dependent characteristics such as, number of metastatic sites, pretreatment with other therapies and BRAF status, confirming that PD1^+^ EVs could be consider general biomarker of innate resistance to immunotherapy with checkpoint inhibitors.

### Assessment of circulating EVs as new biomarkers of resistance to immunotherapy

The following step was the evaluation of these circulating EVs not only as biomarkers of prediction of response to ICI but also directly involved in the mechanism of resistance to anti-PD1 drugs.

We hypothesized that these circulating EVs could reduce the trafficking of immune cells into tumors in non-responders as respect to responders and modify the cytotoxicity of PBMC when nivolumab was added in MM 3D cell culture.

In order to evaluate the trafficking of immune cells to tumor, we generated spheroids from LND1 cells, a BRAF wt MM cell line, to whom we added each of the three different population of PBMCs, isolated from blood of healthy donors, of a responder (RES) and of a non-responder (NRES).

**Fig. 4:**
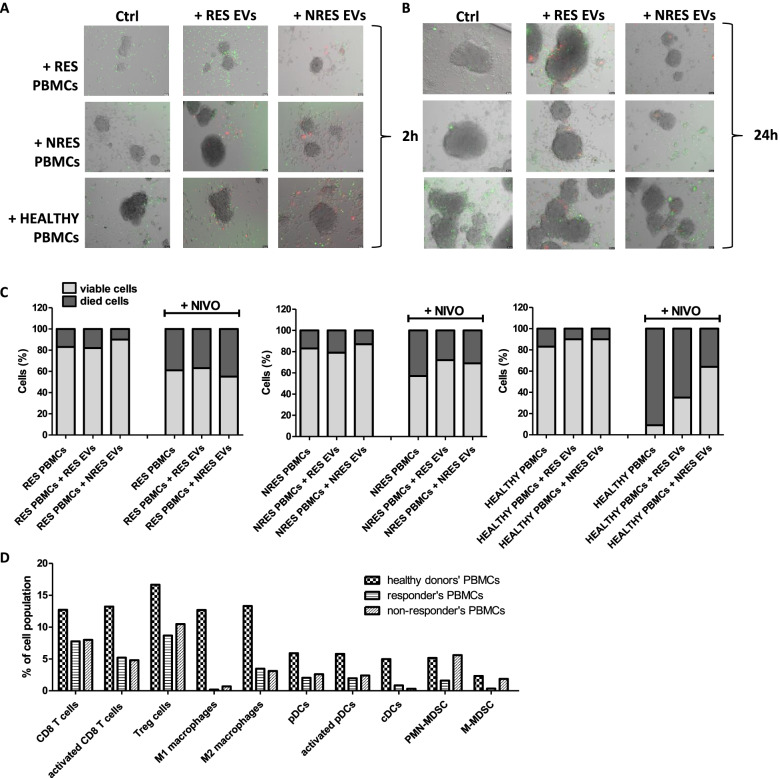
Effect of circulating EVs on immune-cell infiltration and killing into LND1 spheroids. Overview of LND1 spheroid co-cultures with PBMCs of a responder (RES), a non-responder (NRES) or healthy donors (HEALTHY) which were or not preincubated with circulating EVs from the same responder or non-responder. Pictures by fluorescence microscopy were taken after 2 (**A**) and 24 h co-culture (**B**). **C** PBMCs obtained from healthy donors, a responder and a non-responder were preincubated and not with RES EVs or NRES EVs and seeded with LND1 spheroids adding 20μg/ml nivolumab. After 96 h, the viability of tumor cells was evaluated as described in M&M section. **D** FCM characterization of PBMCs obtained from healthy donors, a responder and a non-responder:CD3^+^CD8^+^ T cells, CD3^+^CD8^+^IFNg^+^ activated T cells, CD3^+^CD4^+^CD25^+^FOXP3^+ ^Treg cells, CD14^+^CD68^+^CD80^+^ M1 macrophages, CD14^+^CD68^+^CD206^+^ M2 macrophages, CD11b^+^HLA-DR^+^CD303^+^ pDCs, CD11b^+^HLA-DR^+^CD303^+^CD83^+^ activated pDCs, CD11b^+^HLA-DR^+ ^cDCs, CD15^+^CD14^−^HLA-DR^−^ PMN-MDSC, CD15^−^CD14^+^HLA-DR^−^ M-MDSC

All PBMCs were previously incubated for 1 h with and without circulating EVs (stained in red) isolated from the plasma of the RES and the NRES, in order to simulate the condition of the bloodstream. After 2 h and 24 h, we evaluated the trafficking of PBMCs (stained in green) and as reported in Fig. 4A, after 2 h of incubation, the addition of RES EVs increased the trafficking to tumor of autologous PBMCs while that of NRES EVs seemed to slightly reduce their coming close to the tumor. PBMCs from healthy donors showed greater capacity of trafficking to tumor than those from MM patients; the addition of EVs did not change their behaviour. Circulating EVs did not modify the already reduced ability of NRES PBMCs to reach the tumor. After 24 h, the trafficking of PBMCs to the tumor is more evident; only those from healthy donors show a reduction after the addition of the RES EVs; this is even more evident with the NRES EVs (Fig. 4B).

The evaluation of the killing of MM spheroids by PBMCs of various origins in the presence of nivolumab is reported in Fig. 4C. After 96 h, the anti-PD1 increased the cytotoxicity of PBMCs of about 2.4 folds when PBMCs were isolated from blood of MM patient (RES and NRES). The addition of circulating EVs did not modify the cytotoxic efficacy of RES PBMCs in the presence and absence of nivolumab while it resulted slightly reduced with NRES PBMCs. When MM spheroids were incubated with PBMCs from healthy donors, cell viability was comparable to that found with patients’ PBMCs while nivolumab addition induced a dramatic cell mortality which was reduced in the presence of circulating RES EVs and even more if they were from the non-responder, confirming that the circulating EVs might be directly correlated with the resistance to anti-PD1. These results suggested that PBMCs from MM patients were inherently less responsive to anti-PD1 drugs than those from healthy donors and that the addition of circulating EVs from MM patients reduced ICI efficacy mainly if they derived from non-responders.

In order to investigate the low responsiveness of patients’ PBMCs, we analysed them observing that one thawing step reduced all PBMC populations as respect our previous analysis [25]. However, the PBMCs of MM patients and those of healthy donors differed in number, the main population affected in patients were: i) CD8 T cells, in their totality and the activated ones, and the DCs, both plasmacytoid (pDCs) and conventional (cDCs), which were decreased in patients, ii) the macrophages which were decreased in their totality and more enriched in M2 then M1 population, and iii) the PMN-MDSC and M-MDSC which were, as T reg cells, reduced in responder much more then in non-responder, in agreement with a higher efficacy of ICI in responders (Fig. 4D). All these evidences confirm that the PBMCs of MM patients are less active than those of healthy donors.

**Fig. 5:**
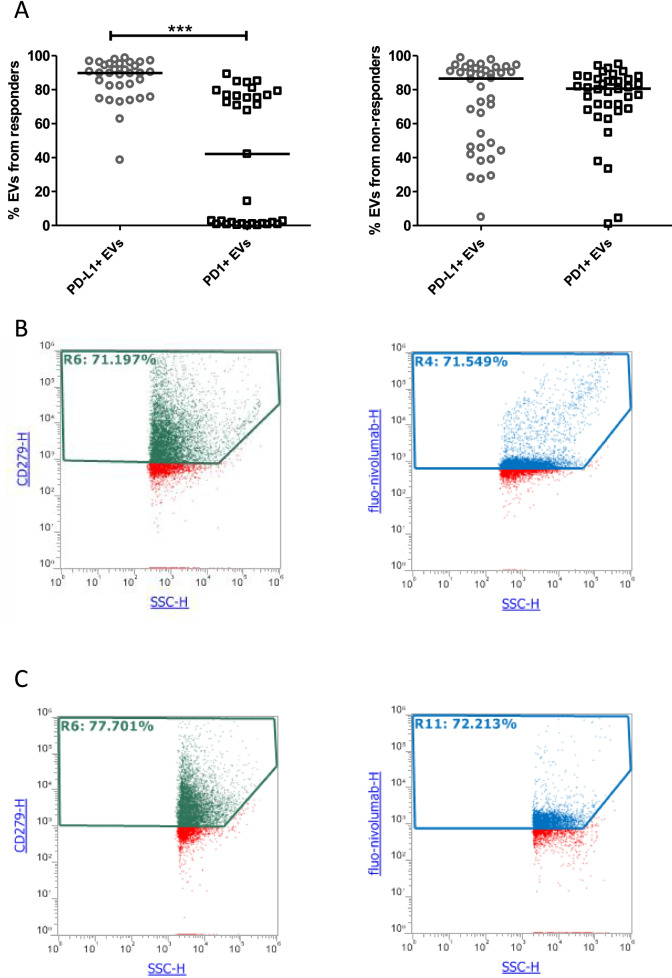
PD1^+^ EVs bind nivolumab. **A** Scatter plots with median of the percentage of PD-L1^+^ and PD1^+^ EVs in plasma of responders (*n* = 38) and non-responders (*n* = 33) (Mann Whitney t test **p < 0.001). **B** Dot plots of the NRES1 EVs resuspended in 1x PBS and positive for PD1 utilising the commercial anti-PD1 (CD279) by eBiosciences (Thermos Fisher) in green and the fluo-nivolumab in blue. **C** Dot plots of the NRES1 EVs resuspended in plasma of healthy donors and positive for PD1 utilising the commercial anti-CD279 in green and the fluo-nivolumab in blue

As shown in Supplemental Fig. S1A, the percentage of positive EVs for PD-L1 in plasma of MM patients was very similar to that of PD1 positive ones. Thus, we determined the concentration of these EVs in responders and non-responders and we observed that in responders the levels of PD-L1^+^ EVs was significantly greater than those of PD1^+^ EVs while the two EV populations were comparable in non-responders (Fig. 5A).

These results suggested evaluating if PD1^+^ EVs were directly correlated with the response to anti-PD1-drugs, by analysing the binding between circulating EVs and the anti-PD1 nivolumab. The possibility of determining the subpopulation of EVs expressing PD1 with commercial anti-PD1 antibodies suggested that these EVs could bind nivolumab. However, we confirmed it by conjugating the anti-PD1 with a fluorescent tag (fluo-nivolumab) and, after incubation with the circulating EVs of 6MM patients, we measured the percentage of PD1^+^ EVs binding the fluorescent drug. The experiments were conducted both in DPBS and in plasma from healthy donors to simulate the administration of nivolumab to patients. In DPBS, the fluo-nivolumab bound circulating EVs with the same efficacy than the commercial anti-PD1 antibody utilised in the EV characterization; in Fig. 5B the dot plots of the non-responder #1 (NRES1) are shown as representative of all. The interaction between EVs and the fluo-nivolumab showed that the efficacy of binding is maintained in the human fluid, too (Fig. 5C).

### Validation of circulating EVs as new biomarkers of resistance to immunotherapy

As reported in Fig. 3B/C, the Cox analyses on PFS revealed PD1^+^ EVs from T cells and of PD-L1^+^ EVs from melanoma as the most significant negative factors while, regarding OS, PD1^+^ EVs from B cells are independent negative prognostic factors.

**Fig. 6:**
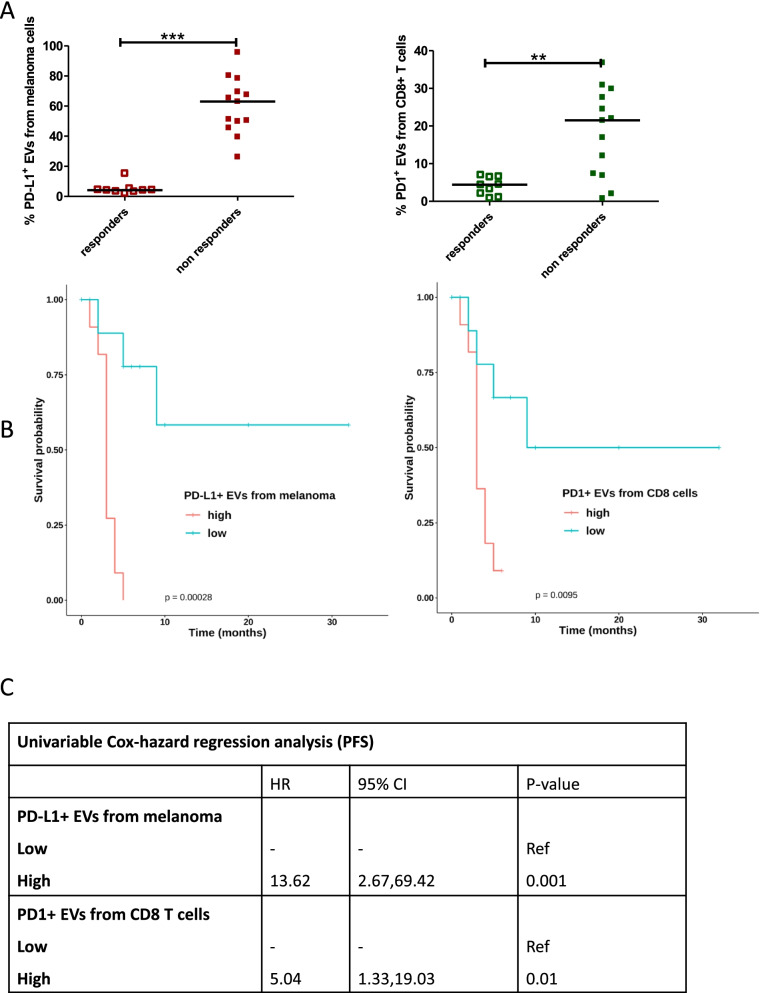
PD-L1^+ ^EVs and PD1^+^ EVs from melanoma and T cells of responders and non-responders enrolled in the validation cohort. **A** Scatter plots with median of the percentage of PD-L1^+^ EVs from melanoma and PD1^+^ EVs from CD8 T cells isolated from responders (9) and non-responders (13) enrolled in the validation independent cohort (Mann Whitney t test **p < 0.05, *** *p* < 0.001). **B** Kaplan–Meier survival curve analysis according to PD-L1^+^ EVs quartiles, with PD-L1^+ ^EVs from melanoma as respect to PFS, and according to PD1^+^ EVs quartiles, with PD1^+ ^EVs from T cells as respect to PFS. **C** Univariable Cox-hazard regression analysis (PFS)

We validated only the first two independent biomarkers because the time elapsed between the analyses and the sampling was too short and did not reach the median OS of the observational study. The validation was carried out by analysing the percentage of PD-L1^+^ EVs from melanoma and PD1^+^ EVs from T cells in the plasma of 9 responders and 13 non-responders prospectively enrolled in the study. Despite the small number of patients enrolled so far, the statistical analysis showed that the levels of the 2 subpopulations of EVs were much higher in non-responders (Fig. 6A), perfectly in agreement with data previously shown in the observational cohort study. The data for PD-L1^+^ EVs from melanoma showed that the statistical power was maintained while it decreased for PD1^+^ EVs from T cells (Fig. 6A). The Kaplan Meier analysis showed that the two independent factors, correlated to the PFS, retained their statistical power (Fig. 6B), despite the small sample size. The univariate analysis confirmed the results as reported in Fig. 6D.

## Discussion

In recent years, liquid biopsies are emerging as a very promising alternative to conventional tissue biopsies for cancer detection, by monitoring tumor progression and response to therapy and tracking tumor evolution. Tumor-derived extracellular vesicles (EVs) arises as an alternative source of biomarkers in liquid biopsies because cancer cells actively produce EVs enriched in cancer-promoting cellular contents, such as immunosuppressive proteins like PD-L1 and PD1, mRNAs and micro-RNAs, which mediate the dysregulation of both the tumour and the protumor immune responses with a consequent establishment of resistance to immunotherapy [16, 45, 46]. The need to identify biomarkers that can allow the selection of MM patients with high probability of response to immunotherapy is imperative and the analysis of the mechanisms involved in determining this lack of response could be of help in identifying other therapeutic strategies that, by blocking this resistance mechanism, can reactivate the immune system.

The analysis of circulating EVs as inhibitors of T cell-mediated immunity and predictors of ICI response has been investigated and found to promote tumor evasion of immune surveillance with a consequent lower response to therapy in other cancer pathology, such as NSCLC, HNSCC, prostate, head and neck, oral-oesophageal, breast and colorectal cancer [16, 47]. Recent studies have hypothesized that both PD-L1 and PD1 positive exosomes or small EVs and soluble forms of PD1 and PD-L1 may play a key role in response to ICI in MM and be predictors of response as well as we recently demonstrated for uPAR^+^ EVs [24, 25, 48].

We have investigated whether circulating small EVs, namely EVs as previously reported, play a role in the response to anti-PD1 and therefore may be predictors of response to this class of drugs. From a large number of MM patients we have isolated circulating EVs and determined the percentage of the PD1^+^ EVs, PD-L1^+^ EVs and PD1^+^PD-L1^+^ EVs, focusing attention on those released by the tumor or by some immune cells, such as CD8^+^ T cells, B cells, monocytes and DCs.

The main results are that: i) both subpopulations of EVs positive for PD1 and PD-L1 were present in high percentages in plasma of MM patients while the double positive are only a small portion of these, ii) if categorized into responders and non-responders, only PD1^+^ EVs and not those positive for PD-L1 were statistically more numerous in non-responders, iii) categorizing these two subpopulations of EVs (positive for PD1 or PD-L1) on their origin (cell type), the level of PD-L1^+^ EVs from melanoma cells and CD8^+^ T cells was higher in non-responders while that of PD1^+^ EVs of any cell origin was always higher in non-responders. A particular situation was found in the subpopulation of responders, where 50% have very low levels (< 10%) of positive EVs for PD1, we investigated if these responders had any particular clinical characteristics but we did not observe any difference statistically significant considering the clinical parameters analysed.

The statistical correlation with clinical outcomes (PFS, OS and ORR) showed that higher levels of these circulating biomarkers also indicate a worse survival. Moreover, the multivariate analysis for PFS showed that high content of PD1^+^ EVs from CD8^+^ T cells and of PD-L1^+^ EVs from melanoma are independent biomarkers thus, of great importance for the selection of patients responsive to ICI. Interestingly, multivariate analysis for OS evidenced the strong correlation between PD1^+^ EVs from B cells and worse OS. Thus, our results demonstrated that selected subpopulation of EVs expressing PD-L1 or PD1 can be valuable tools for oncologists in choosing MM patients with a high probability of responding to immunotherapy with anti-PD1. Finally, we validated our results in a small cohort of MM patients, responders and non-responders, confirming that the two independent factors, correlated with the PFS, maintained the statistical power. The topic on the involvement of circulating EVs in immunotherapy response is still scarcely present in the literature. Chen and collaborators had hypothesized that PD-L1^+^ EVs could be predictive of response to ICI [24]. In our approach, PD-L1^+^ EVs assayed in plasma from a large cohort of MM patients by FCM instead of the ELISA kit did not confirm Chen’s data, however the comparative analysis of these EV levels in responders and non-responders, categorised according to their cells of origin, pointed out that only PD-L1^+^ EVs from melanoma and CD8^+^ T cells were promising biomarkers of resistance to anti-PD1. Another clinical study was conducted by Cordonnier and coauthors who reported that among 46 MM patients, of whom 36 had had anti-PD1 therapy and the others had target therapy, non-responders had higher levels of circulating ExoPD-L1 [48]. Our data confirm Cordonnier’s hypothesis and identify the origin of the PD-L1^+^ EVs population that is higher in non-responders and therefore promising as predictors of response to anti-PD1 therapy.

Accordingly, the multivariate analysis of our data identified three EV subpopulations as independent factors and therefore we can suggest their use to discriminate patients who might respond to anti-PD1 therapy.

It is known that EVs carrying immunosuppressive molecules such as PD-L1, TGF1, FasL, TRAIL, and NKG2D ligands may mediate tumor immune evasion [22]. Then, we investigated whether circulating EVs, including those expressing PD1 and PD-L1, could also be directly responsible for impairing the PD1/PD-L1 checkpoint inhibition by competing with cell surface bound molecules for their binding partners.

We unequivocally demonstrated that the reduced response to anti-PD1 therapy depends on both the intrinsic characteristics of the patients’ immune cells, as the reduction of MDSC and T reg cells in responders [49, 50], and the presence of circulating EVs which reduce the cytotoxic capacity of functioning PBMCs, coming from healthy donors. In fact, PBMCs of a responder and a non-responder showed similar cytotoxic activity than those of healthy donors and their efficacy was not strongly modified by the addition of circulating EVs. The addition of nivolumab strongly increased the efficacy of healthy PBMCs, while slight reactivated those from MM patients. Additionally, healthy PBMCs, previously incubated with circulating EVs of the responder and non-responder, partially lose their aggressiveness. This reduction, that is evident after the addition of EVs from responders, increased dramatically if the vesicles came from the plasma of non-responders. These results is a starting point for the future characterization of the role of various subpopulations of EVs in reducing the response to ICI and suggest that a strategy to reactivate the patients’ PBMCs through EVs elimination could be an add-on therapy enhancing potency of anti-PD1/PD-L1 antibodies [16]. Furthermore, for the first time we demonstrate that circulating EVs bind nivolumab and therefore they are direct neutralizing the therapeutic PD1 antibody. In fact, we observed that the fluo-nivolumab bound circulating EVs of MM patients with quite the same efficiency demonstrated in the characterization of PD1^+^ EVs, both in DPBS and in healthy donors’ EV-depleted plasma.

In the literature there is a controversy between those who suggest EVs as predictors of response to ICI and those who think, like Ugurel, that the use of soluble forms of PD-L1 and PD1 is desirable because the analysis of EVs is elaborate and difficult to standardize. However, differential ultracentrifugation is routinely performed in research labs in which an ultracentrifuge is available [51]. This author demonstrated a direct correlation between baseline serum PD1 and PD-L1 levels and the outcome of anti-PD1 therapy [16]. However, although Ugurel’s study could have the advantage of a rapid analysis of the serum levels of the two biomarkers, the categorization of MM patients into PD1 low and PD-L1 low *vs* PD1 high and/or PD-L1 high selects a small number of patients in the cohort compared to the total enrolled in the study and therefore does not reflect the totality of the responses to anti PD1 therapy. Moreover, in other cancers as in HNSSC and NSCLC, circulating PD-L1 exosomes but not soluble PD-L1 have been found correlated with tumor progression and they justified this evidence with the higher capability of these exosome to bind T cells not only through PD-L1 but also through the major histocompatibility complex expressed on exosomes [52, 53].

## Conclusion

Herein, for the first time we have identified and validated the subpopulations of circulating PD1^+^ EVs and PD-L1^+^ EVs, abundant in bloodstream because cells shed tens of thousands of vesicles per day [54], as promising biomarkers for the response to anti-PD1 therapy. Furthermore, we provided evidences that they are responsible for a reduced efficacy of this treatment acting not only on tumors and close tumor microenvironment but also at distal sites, such as in bloodstream, by both inhibiting immune cells and binding the drug nivolumab. Thus, we provide the rational for using these “selected” subpopulation of circulating EVs for monitoring the response to ICI in metastatic melanoma patients with the great advantage of dosing them in liquid biopsy so a minimally invasive procedure.

## Supplementary Information

**Additional file 1.**

**Additional file 2.**

**Additional file 3.**

## Data Availability

All data and material are available at the laboratory of Experimental Pharmacology of IRCCS Istituto Tumori Giovanni Paolo II. The data regarding the EV characterization presented in this study are openly available in the EV-TRACK knowledgebase [55]. [EV-TRACK ID: EV210207].

## Abbreviations

CR: Complete response
DCR: Disease control rate
DMEM: Dulbecco’s modified Eagle’s medium
EVs: Extracellular vesicles
FBS: Fetal bovine serum
ICI: Immune checkpoint inhibitors
MM: Metastatic melanoma
NRES: Non-responder
ORR: Overall response rate
OS: Overall survival
PFS: Progression free survival
PR: Partial response
RES: Responder
SD: Stable disease
uPAR: Urokinase-type Plasminogen Activator Receptor
NLR: Neutrophil to lymphocyte ratio
PLR: Platelet to lymphocyte ratio
