# Supplementary material for: Circulating extracellular vesicles expressing PD1 and PD-L1 predict response and mediate resistance to checkpoint inhibitors immunotherapy in metastatic melanoma
Source: Mol Cancer. 2022 Jan 18;21:20. doi: 10.1186/s12943-021-01490-9 (PMC8764806; doi:10.1186/s12943-021-01490-9)
Supplement: Supplementary file 1 — Additional file 1. [file 12943_2021_1490_MOESM1_ESM.pdf]

Supplemental Figures

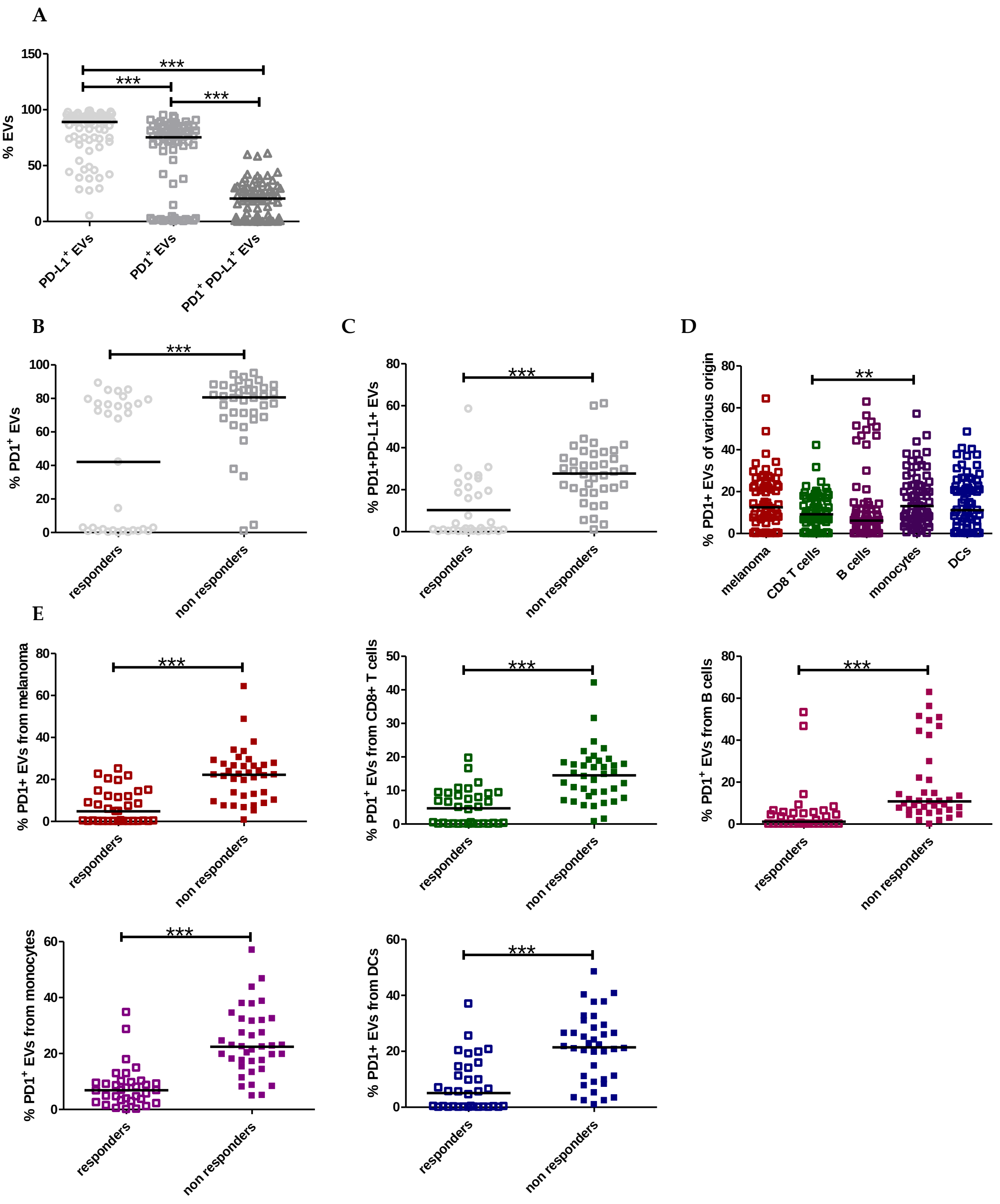

Fig. S1 Circulating PD1+ EVs derived from different cell types released clustered by response to therapy. Scatter plot with median of A. the percentage of PD-L1+ EVs, PD1+ EVs and PD1+ PD-L1+ EVs in plasma of 71 MM patients; B. the percentage of PD1+ EVs from responders (n = 38) and non-responders (n = 33); C. the percentage of PD1+ PD-L1+ EVs from responders (n = 38) and non-responders (n = 33); D. the percentage of PD1+ EVs of different origin isolated in plasma of all 71 MM patients and E. the percentage of PD1+ EVs of different origin from responders (n=38) and non-responders (n=33). (Mann Whitney t test *p < 0.05, *** p < 0.001).

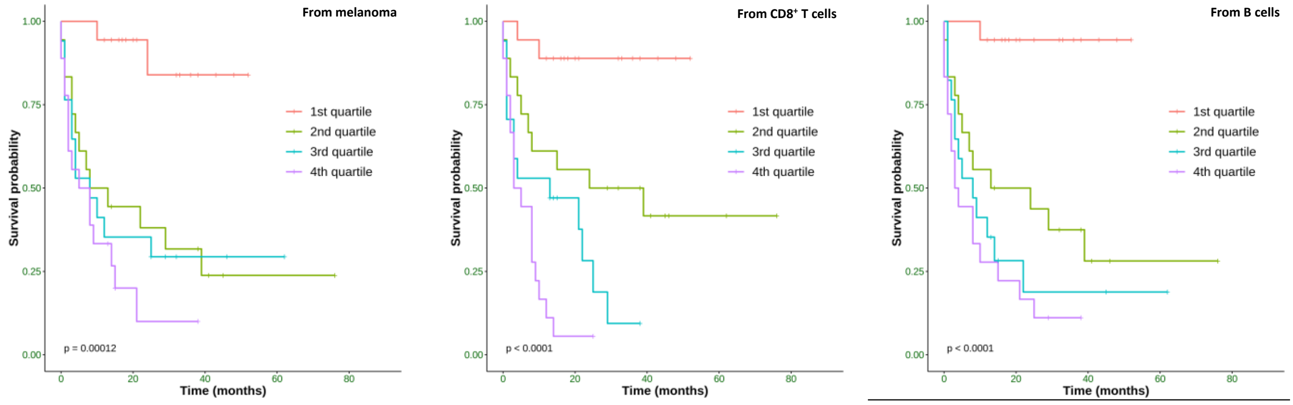

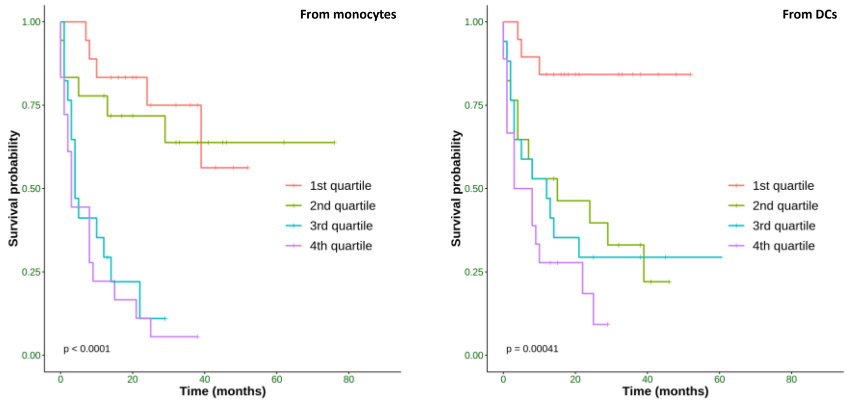

From melanoma	1st quartile	2nd quartile	3rd quartile
2nd quartile	0.00110	-	-
3rd quartile	0.00055	0.82154	-
4th quartile	2.7e-06	0.14601	0.32208
From CD8 T cells			
2nd quartile	0.02354	-	-
3rd quartile	0,00025	0.05934	-
4th quartile	5.8e-07	0.00239	0.06412
From B cells			
2nd quartile	0.00025	-	-
3rd quartile	5.5e-06	0.30390	-
4th quartile	3.4e-07	0.04718	0.42876
From monocytes			
2nd quartile	0.72723	-	-
3rd quartile	7.3e-05	0.00263	-
4th quartile	4.3e-06	0.00019	0.44963
From DCs			
2nd quartile	0.0034	-	-
3rd quartile	0.0018	0.8546	-
4th quartile	3.3e-05	0.1124	0.2455

Fig. S2 Evaluation of OS in patients with PD1^{+} EVs derived from melanoma cells and immune cells. Kaplan–Meier survival curve analysis according to PD1^{+} EVs quartiles, with these EVs from melanoma cells, CD8^{+} T cells, B cells, monocytes and DCs as respect to OS. For each analysis, a pairwise comparison of curves has been performed with p-values reported in tables next to each graph.

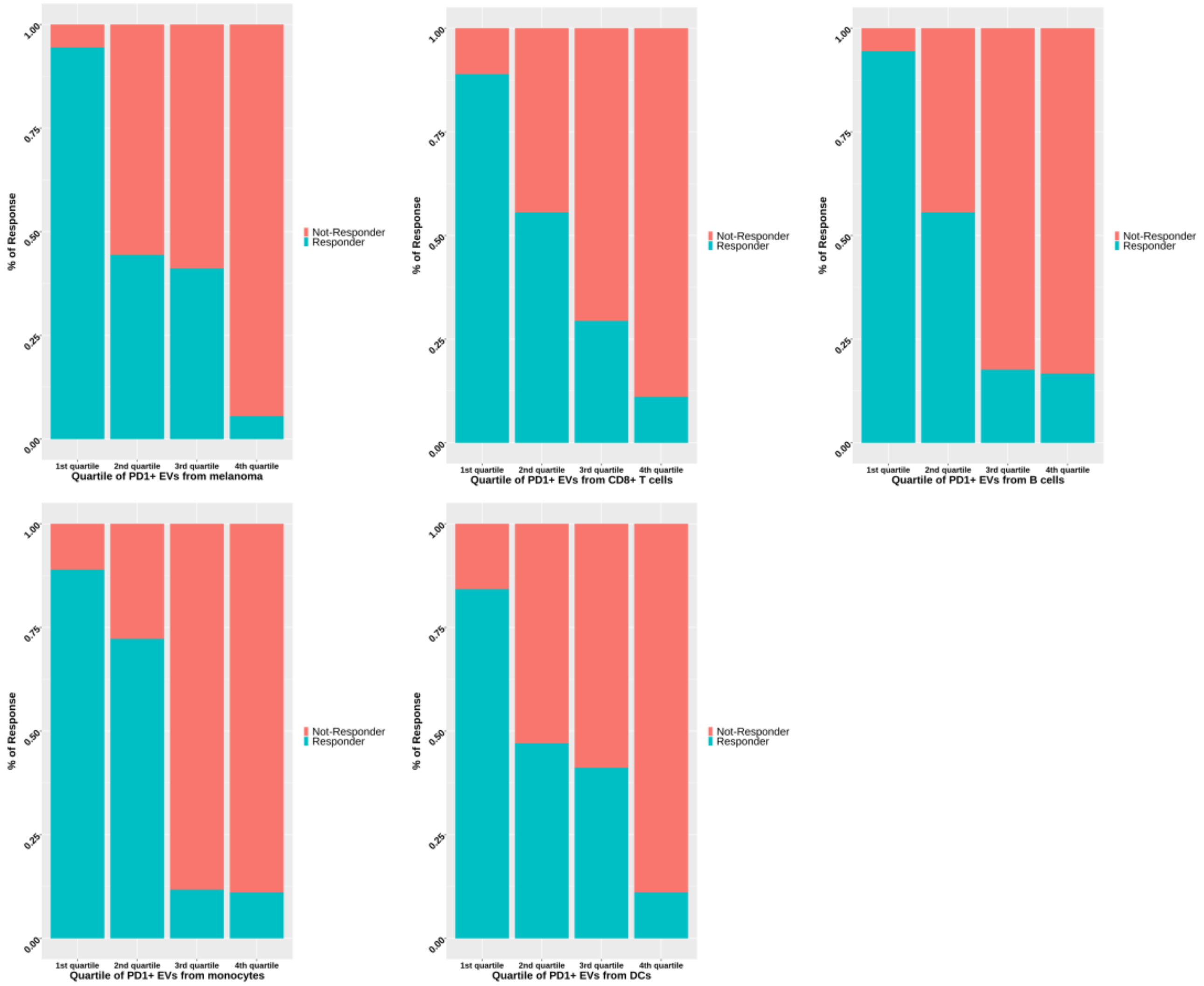

Fig. S3 Evaluation of ORR analysis in patients with PD1⁺ EVs. The distribution of the best responses stratifying patients by PD1⁺ EVs in responder and non-responders and coming from melanoma cells, CD8⁺ T cells, B cells, monocytes and DCs.

Characteristic	N	HR[1]	95% CI[1]	p-value
PD1_EVs_from_melanoma	71			
1st quartile		—	—	
2nd quartile		2.63	1.09, 6.35	0.032
3rd quartile		3.41	1.44, 8.09	0.005
4th quartile		6.97	2.92, 16.6	<0.001
PD1_EVs_from_CD8_Tcells	71			
1st quartile		—	—	
2nd quartile		1.96	0.80, 4.80	0.14
3rd quartile		4.52	1.90, 10.7	<0.001
4th quartile		6.79	2.86, 16.1	<0.001
PD1_EVs_from_B_cells	71			
1st quartile		—	—	
2nd quartile		2.45	1.01, 5.93	0.046
3rd quartile		4.55	1.90, 10.9	<0.001
4th quartile		5.69	2.42, 13.4	<0.001
PD1_EVs_from_monocytes	71			
1st quartile		—	—	
2nd quartile		1.13	0.46, 2.79	0.79
3rd quartile		5.47	2.38, 12.5	<0.001
4th quartile		5.11	2.29, 11.4	<0.001
PD1_EVs_from_DCs	71			

Characteristic	N	HR[1]	95% CI[1]	p-value
1st quartile		—	—	
2nd quartile		2.39	1.02, 5.60	0.045
3rd quartile		2.64	1.13, 6.22	0.026
4th quartile		6.25	2.74, 14.2	<0.001
PDL1_EVs_from_DCs	71			
1st quartile		—	—	
2nd quartile		1.66	0.75, 3.69	0.21
3rd quartile		1.82	0.82, 4.07	0.14
4th quartile		1.88	0.86, 4.11	0.11
PDL1_EVs_from_melanoma	71			
1st quartile		—	—	
2nd quartile		1.88	0.81, 4.36	0.14
3rd quartile		2.06	0.90, 4.70	0.088
4th quartile		4.06	1.85, 8.91	<0.001
PDL1_EVs_from_CD8_Tcells	71			
1st quartile		—	—	
2nd quartile		1.40	0.62, 3.19	0.42
3rd quartile		2.07	0.92, 4.63	0.077
4th quartile		2.30	1.07, 4.94	0.033
PDL1_EVs_from_B_cells	71			
1st quartile		—	—	

Characteristic	N	HR[1]	95% CI[1]	p-value
2nd quartile		1.29	0.58, 2.84	0.53
3rd quartile		2.08	0.95, 4.55	0.065
4th quartile		1.37	0.61, 3.06	0.44
PDL1_EVs_from_monocytes	71			
1st quartile		—	—	
2nd quartile		0.97	0.46, 2.08	0.95
3rd quartile		1.21	0.57, 2.57	0.63
4th quartile		0.80	0.37, 1.77	0.59
uPAR_EVs_from_melanoma	71			
1st quartile		—	—	
2nd quartile		2.17	0.88, 5.32	0.092
3rd quartile		4.76	2.01, 11.3	<0.001
4th quartile		6.70	2.81, 16.0	<0.001
uPAR_EVs_from_CD8_Tcells	71			
1st quartile		—	—	
2nd quartile		1.76	0.74, 4.20	0.20
3rd quartile		3.07	1.34, 7.06	0.008
4th quartile		5.01	2.18, 11.5	<0.001
uPAR_EVs_from_B_cells	71			
1st quartile		—	—	
2nd quartile		2.43	1.12, 5.29	0.025

Characteristic	N	HR[1]	95% CI[1]	p-value
3rd quartile		1.96	0.87, 4.39	0.10
4th quartile		1.69	0.76, 3.78	0.20
uPAR_EVs_from_monocytes	71			
1st quartile		—	—	
2nd quartile		2.24	1.04, 4.85	0.040
3rd quartile		2.76	1.24, 6.12	0.013
4th quartile		0.88	0.37, 2.07	0.77
uPAR_EVs_from_DCs	71			
1st quartile		—	—	
2nd quartile		1.26	0.55, 2.87	0.59
3rd quartile		1.73	0.77, 3.87	0.19
4th quartile		3.28	1.51, 7.17	0.003
BRAF_status	68			
mutated		—	—	
wild-type		0.85	0.49, 1.48	0.56
Previous_therapy_for_metastatic_disease	71			
No		—	—	
Yes		1.02	0.59, 1.75	0.95
Sex	71			
female		—	—	
male		1.09	0.64, 1.87	0.75
PLR	71			

Characteristic	N	HR[1]	95% CI[1]	p-value
Low		—	—	
High		0.86	0.48,1.52	0.61
NLR	71			
Low		—	—	
High		1.03	0.58,1.82	0.91

[1]HR = Hazard Ratio, CI = Confidence Interval

***Fig. S4 Univariate Cox-hazard regression analysis for PFS*.**

Characteristic	N	HR[1]	95% CI[1]	p-value
PD1_EVs_from_melanoma	71			
1st quartile		—	—	
2nd quartile		9.18	2.07, 40.8	0.004
3rd quartile		10.0	2.24, 44.9	0.003
4th quartile		15.6	3.54, 68.8	<0.001
PD1_EVs_from_CD8_Tcells	71			
1st quartile		—	—	
2nd quartile		5.48	1.20, 25.2	0.029
3rd quartile		11.9	2.67, 53.1	0.001
4th quartile		21.2	4.81, 93.9	<0.001
PD1_EVs_from_B_cells	71			
1st quartile		—	—	
2nd quartile		15.9	2.06, 122	0.008
3rd quartile		24.1	3.13, 185	0.002
4th quartile		33.8	4.46, 256	<0.001
PD1_EVs_from_monocytes	71			
1st quartile		—	—	
2nd quartile		1.32	0.40, 4.34	0.64
3rd quartile		6.46	2.26, 18.5	<0.001
4th quartile		8.53	3.07, 23.7	<0.001
PD1_EVs_from_DCs	71			

Characteristic	N	HR[1]	95% CI[1]	p-value
1st quartile		—	—	
2nd quartile		5.78	1.63, 20.5	0.007
3rd quartile		6.19	1.74, 22.0	0.005
4th quartile		10.3	2.95, 35.7	<0.001
PDL1_EVs_from_DCs	71			
1st quartile		—	—	
2nd quartile		1.35	0.52, 3.51	0.54
3rd quartile		1.88	0.75, 4.67	0.18
4th quartile		1.99	0.83, 4.75	0.12
PDL1_EVs_from_melanoma	71			
1st quartile		—	—	
2nd quartile		5.10	1.40, 18.6	0.013
3rd quartile		6.04	1.70, 21.4	0.005
4th quartile		10.6	3.10, 36.5	<0.001
PDL1_EVs_from_CD8_Tcells	71			
1st quartile		—	—	
2nd quartile		1.79	0.64, 5.06	0.27
3rd quartile		3.41	1.28, 9.12	0.015
4th quartile		3.18	1.23, 8.23	0.017
PDL1_EVs_from_B_cells	71			
1st quartile		—	—	

Characteristic	N	HR[1]	95% CI[1]	p-value
2nd quartile		1.74	0.67, 4.49	0.25
3rd quartile		2.49	0.98, 6.32	0.056
4th quartile		2.09	0.82, 5.30	0.12
PDL1_EVs_from_monocytes	71			
1st quartile		—	—	
2nd quartile		1.25	0.54, 2.89	0.61
3rd quartile		1.16	0.49, 2.74	0.73
4th quartile		0.84	0.34, 2.07	0.70
uPAR_EVs_from_melanoma	71			
1st quartile		—	—	
2nd quartile		15.7	2.04, 121	0.008
3rd quartile		24.8	3.26, 189	0.002
4th quartile		35.0	4.57, 268	<0.001
uPAR_EVs_from_CD8_Tcells	71			
1st quartile		—	—	
2nd quartile		6.95	1.54, 31.4	0.012
3rd quartile		11.5	2.60, 50.5	0.001
4th quartile		13.8	3.12, 61.0	<0.001
uPAR_EVs_from_B_cells	71			
1st quartile		—	—	
2nd quartile		3.28	1.25, 8.57	0.016

Characteristic	N	HR[1]	95% CI[1]	p-value
3rd quartile		2.80	1.03, 7.60	0.043
4th quartile		2.54	0.93, 6.88	0.068
uPAR_EVs_from_monocytes	71			
1st quartile		—	—	
2nd quartile		1.76	0.78, 4.00	0.17
3rd quartile		1.42	0.59, 3.43	0.44
4th quartile		0.80	0.31, 2.02	0.63
uPAR_EVs_from_DCs	71			
1st quartile		—	—	
2nd quartile		1.98	0.68, 5.81	0.21
3rd quartile		3.33	1.18, 9.39	0.023
4th quartile		3.64	1.31, 10.2	0.014
BRAF_status	68			
mutated		—	—	
wild-type		0.69	0.37, 1.29	0.24
Previous_therapy_for_metastatic_disease	71			
No		—	—	
Yes		1.50	0.81, 2.76	0.19
Sex	71			
female		—	—	
male		1.03	0.56, 1.89	0.93
PLR	71			

Characteristic	N	HR[1]	95% CI[1]	p-value
Low		—	—	
High		1	0.98,1.01	0.97
NLR	71			
Low		—	—	
High		1.02	0.53,1.92	0.93

[1]HR = Hazard Ratio, CI = Confidence Interval

Fig. S5 Univariate Cox-hazard regression analysis for OS.

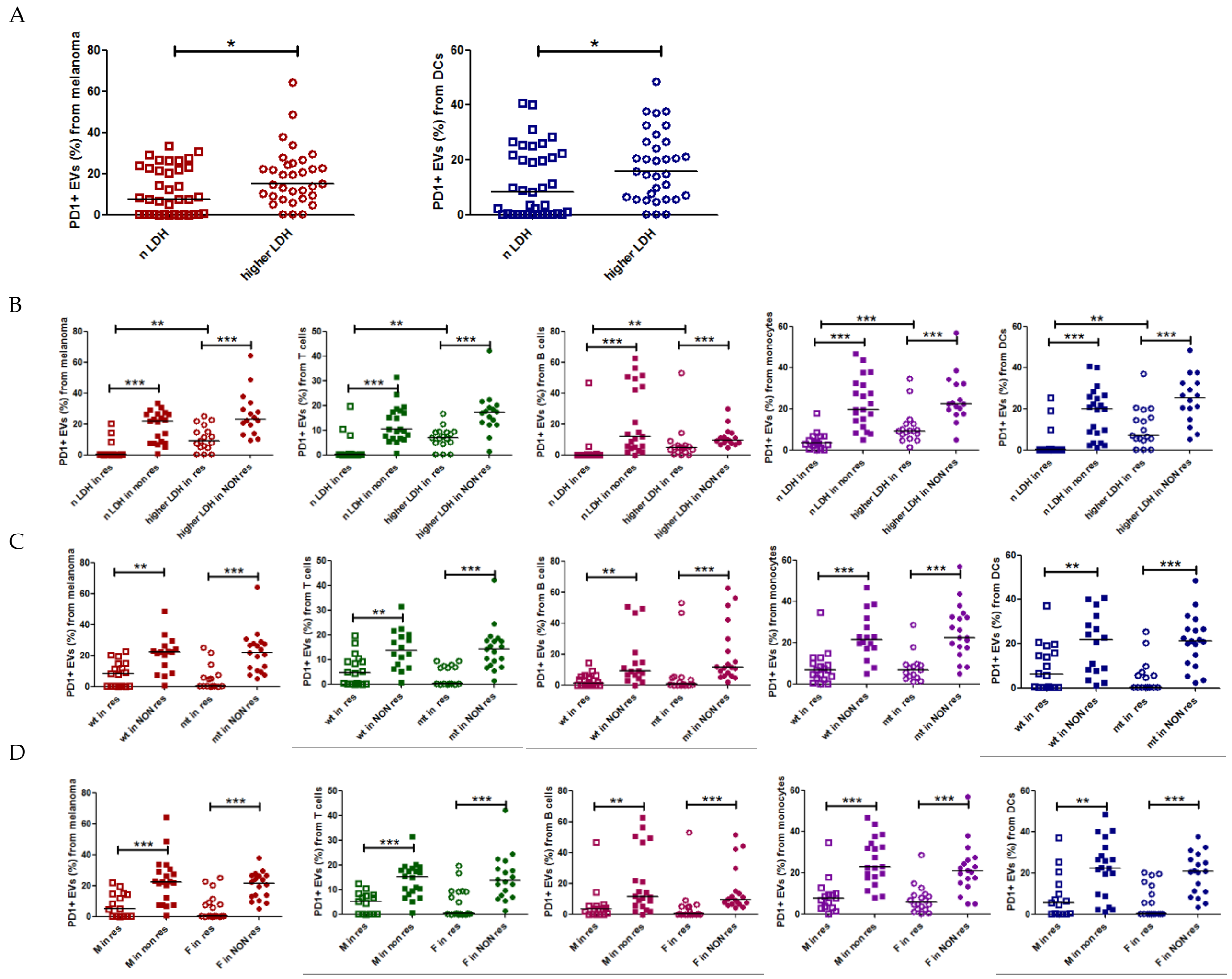

Fig. S6. Correlation between PD1+ EVs and clinical features.. Scatter plots with median showing A. the percentage of PD1⁺ EVs from melanoma cells and DCs in MM patients with normal and higher LDH and the percentage of PD1⁺ EVs of different origin from responders and non-responders B. with normal and higher LDH, C. in BRAF mutated patients *vs* BRAF wt, D. in function of gender,. (Mann Whitney t test * $p < 0.05$, ** $p < 0.01$, *** $p < 0.001$).

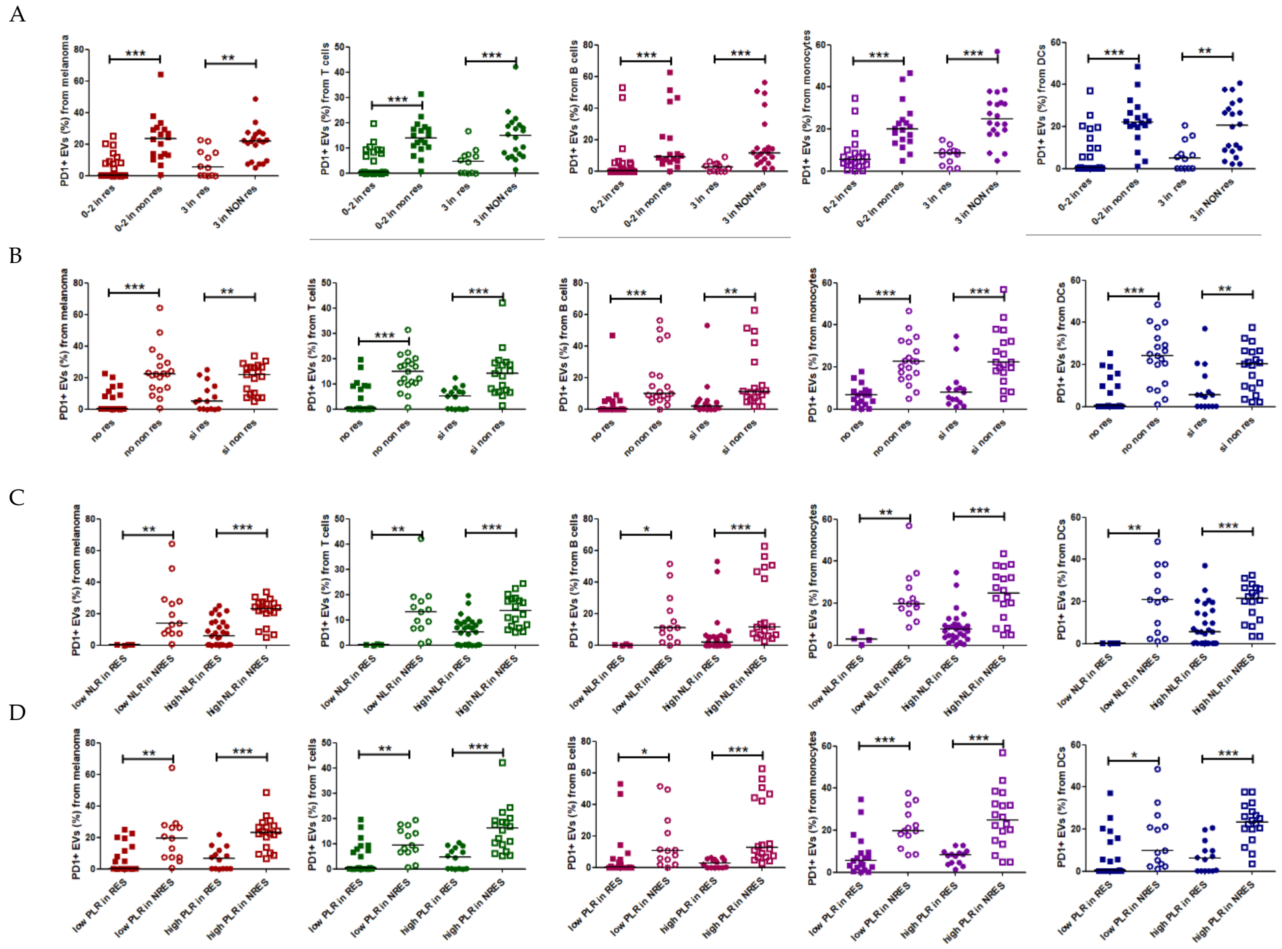

Fig. S7. Correlation between PD1+ EVs and clinical features. Scatter plots with median showing A. the percentage of PD1+ EVs of various cell origin A. in MM patients in function the number of metastatic site, B. in naïve *vs* pretreated MM patients, in function of C. NLR and D. PLR. (Mann Whitney t test * $p < 0.05$, ** $p < 0.01$, *** $p < 0.001$).
