# Supplementary material for: Circulating extracellular vesicles expressing PD1 and PD-L1 predict response and mediate resistance to checkpoint inhibitors immunotherapy in metastatic melanoma
Source: Mol Cancer. 2022 Jan 18;21:20. doi: 10.1186/s12943-021-01490-9 (PMC8764806; doi:10.1186/s12943-021-01490-9)
Supplement: Supplementary file 2 — Additional file 2. [file 12943_2021_1490_MOESM2_ESM.pdf]

**Supplemental M&M**

**M&M S1.**

**EVs analysis by Flow Cytometry.**

All experiments were carried out utilising the Attune NxT Flow Cytometer (Thermo Fisher) configured with 4 spatially separated lasers, red, green, yellow and violet. For EV analysis, we utilised a specific filter for small particles (Attune™ NxT OD2-488/10 Filter) and for the setup, we followed Gorgun's protocol [33]. All solutions were filtered with 0.22-μm filter.

In details, the EV size-based setup was done utilising two different mix of fluorescent beads of varied diameters (from 0.1 to 1 μm), the Megamix-Plus SSC (Biocytex) and a mix (1:1) of Megamix-Plus SSC (Biocytex) and Megamix-Plus FCS (Biocytex). The physical parameters were: FSC: 80 and SSC: 220. The threshold was on FSC (0.1 x 1000). Speed of analysis 12.5μl/min.

The FCM analysis allows to identify the region (gate 14/19) where nanoparticles from 100 to 900nm are.

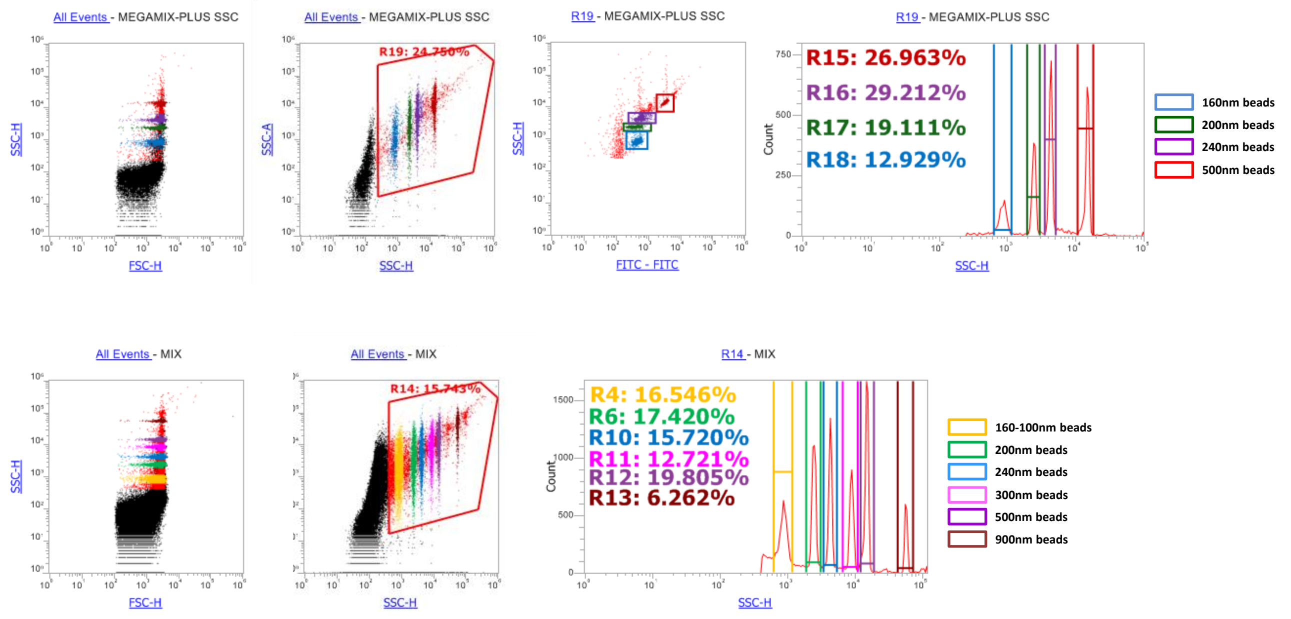


Antibodies were filtrated utilizing 0.45μm-centrifuge tube filters (Corning) to exclude antibody aggregates [doi.org/10.1002/cyto.a.22649].

During the multicolour FCM, the setup of all the fluorescences was made during the compensation procedure with the Fluorescence Minus One (FMO) control, following the instrument instruction.

We utilised 0.22nm-filtered DPBS as the Blank buffer control.

To evaluate the impact that proteins aggregates has on the analysis, EV samples were incubated with 0.05% Triton X-100 and roughly vortexed. The analysis of the autofluorescence of unstained samples with and without the detergent showed the same readout confirming that the unstained samples are protein aggregates-free EV population and that their autofluorescence can be considered to gate the region where the positive events have to be collected.

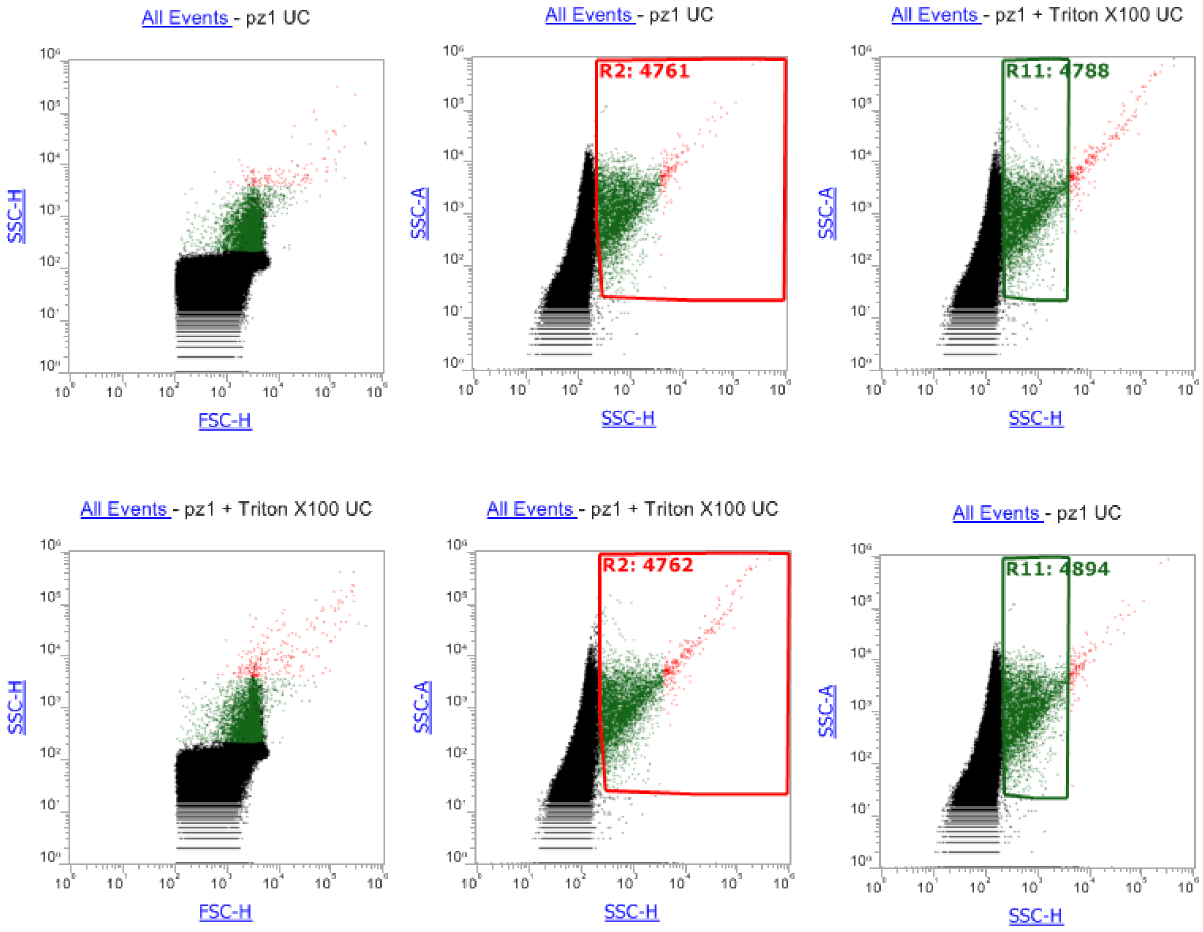


The analysis of each EV sample was carried out by incubating EVs with or without the specific fluorescent primary antibodies and for each sample, we collected 10.000 events of the population gated in the dot plot SSC-A *vs* SSC-H.

**EV gating strategy**

For each patient, we determined all circulating EVs positive for at least one of the three tetraspanins (CD9, CD63 and CD81). The data were analysed in dotplots showing the double positivity of EVs for CD9/CD63,

CD9/CD81 and CD63/CD81. Our analysis showed 2 different experimental situations, described below. Since the expression of the three tetraspanins varies from patient to patient, in the description of the two experimental situations we found in the study, we will generically call them CDx, CDy and CDz to explain the EV gate strategy applied.

The former experimental situation:

- All EVs positive for CDx were also positive for CDy and not vice versa; it means that the $CDy^+$ EVs also include all the positive ones for CDx.
- all EVs positive for the CDy are also positive for the CDz and not vice versa, it means that all the $CDz^+$ EVs also include the CDy positive ones.

Thus, we will consider in the following characterizations only the **$CDz^+$ EVs** because also inclusive of EVs positive for CDx and CDy.

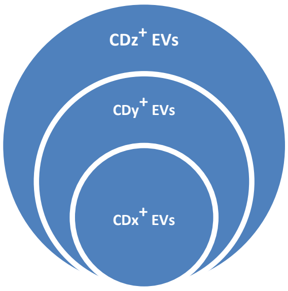


The latter experimental situation:

- All $CDx^+$ EVs are also positive for CDy and not vice versa, thus $CDy^+$ EVs include $CDx^+$ EVs.
- Not all $CDy^+$ EVs are positive for CDz and not all $CDz^+$ EVs are positive for CDy.

Thus: we considered the sum: **$CDy^+CDz^-$ EVs + $CDy^-CDz^+$ EVs + $CDy^+CDz^+$ EVs**
