# Supplementary material for: Circulating extracellular vesicles expressing PD1 and PD-L1 predict response and mediate resistance to checkpoint inhibitors immunotherapy in metastatic melanoma
Source: Mol Cancer. 2022 Jan 18;21:20. doi: 10.1186/s12943-021-01490-9 (PMC8764806; doi:10.1186/s12943-021-01490-9)
Supplement: Supplementary file 3 — Additional file 3. [file 12943_2021_1490_MOESM3_ESM.pdf]

Supplemental Tables

Table S1. Main clinical characteristics of MM patients enrolled in the validation cohort study (22)

**Median age at metastatic disease (range)**	**67 (41-92) years**
**Sex, n (%)**	
male	14 (63.6)
female	8 (36.4)
**Type of melanoma, n (%)**	
cutaneous	16 (72.7)
Uveal	2 (9.1)
Unknown origin	4 (18.2)
**BRAF Status , n (%)**	
mutated	5 (22.7)
wt	17 (77.3)
**Previous systemic therapy for metastatic disease, n (%)**	
yes	6 (27.3)
no	16 (72.7)
**Stage at metastatic disease *, n (%)**	
M1a	5 (22.7)
M1b	3 (13.7)
M1c	9 (40.9)
M1d	5 (22.7)
**N. of metastatic sites*, n (%)**	
<3	11 (50)
≥3	11 (50)
**LDH , n (%)**	
<ULN	11 (50)
>ULN	9 (40.9)
Unspecified	2 (9.1)
**Best response, n (%)**	
ORR	9 (40.9)
DCR	9 (40.9)
CR	1 (4.5)
PD	13 (59.1)
**PFS median, months**	3.5

Table S2. **Median values of PD1+ EVs released from melanoma cells or immune cells as a function of the response to immunotherapy**

PD1⁺ EVs released from	nLDH		Higher LDH	
Melanoma	7.47		14.95	
DCs	8.32		15.88	
	res	Non res	res	Non res
melanoma	0.094	21.84	8.95	23.23
CD8 T cells	0.083	10.42	6.87	17.13
B cells	0.052	11.78	4.49	9.51
Monocytes	3.43	19.79	9.17	22.43
DCs	0.18	19.88	7.01	5.27
**PD1⁺ EVs released from**	**BRAF wt**		**BRAF (V600E)**	
	res	Non res	res	Non res
melanoma	8.17	22.27	0.31	21.82
CD8 T cells	4.71	13.55	0.26	14.24
B cells	0.98	8.98	0.51	11.46
Monocytes	6.75	21.54	6.94	22.45
DCs	6.07	21.74	0.30	21.09
**PD1⁺ EVs released from**	**Male**		**Female**	
	res	Non res	res	Non res
melanoma	4.87	22.40	0.37	21.57
CD8 T cells	5.06	15.12	0.46	13.66
B cells	3.47	11.40	0.07	9.51
Monocytes	7.74	22.86	5.76	20.82
DCs	5.57	22.55	0.31	20.83
**PD1⁺ EVs released from**	**Metastatic sites ≤ 2**		**Metastatic sites > 3**	
	res	Non res	res	Non res
melanoma	0.42	23.64	5.38	22.04
CD8 T cells	0.46	13.96	4.72	14.82
B cells	0.10	9.25	2.74	11.62
Monocytes	5.63	20.00	8.54	24.70
DCs	0.37	22.06	5.11	20.49
**PD1⁺ EVs released from**	**naive**		**pretreated**	
	res	Non res	res	Non res
melanoma	0.37	22.28	4.87	21.82
CD8 T cells	0.46	14.84	5.06	14.24
B cells	0.07	9.78	2.00	10.93
Monocytes	6.75	22.75	8.04	22.41
DCs	0.36	24.08	5.57	20.29
**PD1⁺ EVs released from**	**Low NLR**		**High NLR**	
	res	Non res	res	Non res
melanoma	0.19	13.76	5.88	23.23
CD8 T cells	0.16	13.08	5.06	13.76
B cells	0.06	10.93	2.00	11.62
Monocytes	2.87	19.79	7.74	24.68
DCs	0.18	20.95	5.60	21.51
**PD1⁺ EVs released from**	**Low PLR**		**High PLR**	
	res	Non res	res	Non res
melanoma	0.42	19.62	6.62	23.23

CD8 T cells	0.46	9.44	4.72	16.12
B cells	0.10	10.79	2.77	12.62
Monocytes	5.63	19.79	8.30	24.68
DCs	0.37	9.88	6.10	23.43
